# Supplementary material for: Analysis of PPARγ Signaling Activity in Psoriasis
Source: Int J Mol Sci. 2021 Aug 10;22(16):8603. doi: 10.3390/ijms22168603 (PMC8395241; doi:10.3390/ijms22168603)
Supplement: Supplementary file 1 [file ijms-22-08603-s001.zip › Supplemental materials_Analysis of PPARg signaling activity in psoriasis/Pathway models/Models images and html files/Differentiation of psoriatic T cells/6332.html]

IL6


# Protein IL6

|  |  |
| --- | --- |
| URN | urn:agi-llid:3569 |
| Total Entities | 0 |
| Connectivity | 23636 |
| Name | IL6 |
| Description | interleukin 6 |
| Notes | This gene encodes a cytokine that functions in inflammation and the maturation of B cells. In addition, the encoded protein has been shown to be an endogenous pyrogen capable of inducing fever in people with autoimmune diseases or infections. The protein is primarily produced at sites of acute and chronic inflammation, where it is secreted into the serum and induces a transcriptional inflammatory response through interleukin 6 receptor, alpha. The functioning of this gene is implicated in a wide variety of inflammation-associated disease states, including suspectibility to diabetes mellitus and systemic juvenile rheumatoid arthritis. Alternative splicing results in multiple transcript variants. [provided by RefSeq, Dec 2015] |
| Primary Cell Localization | Extracellular |
| Class | Ligand |
| ObjectType | Protein |

---

|  |  |
| --- | --- |
| Pathway | Alzheimer's Disease |
|  | Androgen Deficiency in Male Obesity |
|  | Cortisol in Resolving Inflammation |
|  | Toll-like Receptors Act through MYD88-TIRAP Signaling |
|  | TLR4 Signaling in Leukocytes |
|  | Dectin-2 (CLEC6A)/Mincle (CLEC4E)/BDCA2 (CLEC4C) Signaling |
|  | T-Cell Maturation (Hypothesis) |
|  | AHR Signaling in Th17 Cells Function |
|  | Th17-Cell Differentiation |
|  | Platelet Activation via Adhesion Molecules |
|  | Proplatelet Maturation |
|  | Toll-like Receptors in Sterile Inflammation |
|  | Toll-like Receptor Independent Sterile Inflammation |
|  | Hypothalamic-Pituitary-Adrenal Axis in Acute Phase Response |
|  | Annexins in Inflammation Resolution |
|  | Lipids Enhance Apoptotic Cell Engulfment and Reduce Inflammation |
|  | Keep out Signal: LTF Inhibits Neutrophil Recruitment and Inflammation |
|  | Basophil Activation |
|  | Eosinophil Survival by Cytokine Signaling |
|  | Eosinophil Activation |
|  | Mast-Cell Activation via IgE Signaling |
|  | Mast-Cell Activation without Degranulation |
|  | Mast-Cell Activation without Degranulation through KIT/F2R Signaling |
|  | Mast-Cells De Novo Synthesized Mediators via IgE Independent Signaling |
|  | Mast-Cell Activation without Degranulation through IL1R1 and TLR Signaling |
|  | Macrophage Related Iron Uptake and Release |
|  | Macrophage M1 Lineage |
|  | AHR Signaling in M1 Macrophages Function |
|  | AXL Receptor Inhibits Macrophages and Dendritic Cells Function |
|  | Negative Acute Phase Proteins Synthesis |
|  | Positive Acute Phase Proteins Synthesis |
|  | Vascular Endothelial Cell Activation by Cytokines |
|  | Vascular Endothelial Cell Activation by Blood Coagulation Factors |
|  | Adipokines Production by Adipocyte |
|  | Nociception Expression Targets Signaling |
|  | ADRA1A -> IL6 Production |
|  | BDKRB1/2 -> Interleukins Production |
|  | CNR1/2 -> IL1B/2/4/6/10 Production |
|  | PTGIR -> IL6 Production |
|  | PTGER2/3 -> Inflammation-Related Expression Targets |
|  | HTR1 -> IL6 Production |
|  | HTR7 -> IL6 Production |
|  | TACR1 -> TNF/IL6/IL8 Production |
|  | IL6 Expression Targets -> Nociception |
|  | IL1B Expression Targets -> Nociception |
|  | Leptin -> CD25/IL6/IL10 Production |
|  | Proteins Involved in Atherosclerosis |
|  | Dendritic Cells Function in Atherosclerosis |
|  | RAGE/AGER and S100 Proteins in Cardiovascular Injury |
|  | Proteins Involved in Dilated Cardiomyopathy |
|  | Proteins Involved in Hypertrophic Cardiomyopathy |
|  | Cardiomyocyte Hypertrophy |
|  | Hypertrophic Cardiomyopathy |
|  | Proteins Involved in Arterial Hypertension |
|  | Arterial Hypertension |
|  | Proteins Involved in Pulmonary Hypertension |
|  | Proteins Involved in Myocardial Ischemia |
|  | Lymphocyte Mediated Myocardial Injury in Myocarditis |
|  | Proteins Involved in Myocarditis |
|  | SOCS in Negative Feedback Regulation in Myocarditis |
|  | Toll-like Receptors (TLR) in Antiviral Innate Immune Response |
|  | Hair Follicle Keratinocyte Apoptosis |
|  | Androgens Promote Scalp Dermal Papilla Regression |
|  | Proteins Involved in Melanoma |
|  | Proteins Involved in Psoriasis |
|  | T-Cells Differentiation Block in Psoriasis |
|  | Dendritic Cells Function in Psoriasis |
|  | Proteins Involved in Photoaging |
|  | Dendritic Cell Activation in Systemic Lupus Erythematosis |
|  | Proteins Involved in Systemic Lupus Erythematosus |
|  | Th17-Cell Function in Systemic Lupus Erythematosus |
|  | Neutrophil and Macrophage Function in Systemic Lupus Erythematosus |
|  | Proteins Involved in Vitiligo |
|  | Proteins with Altered Expression in Vitiligo |
|  | Vitiligo |
|  | T-Cell Cytotoxic Response against Melanocytes in Vitiligo |
|  | Humoral Immunity in Vitiligo |
|  | Proteins Involved in Diabetes Mellitus Type 1 |
|  | Proteins Involved in Diabetes Mellitus Type 2 |
|  | Tubular Cell Dysfunction in Progressive Diabetic Nephropathy |
|  | Endothelial Cell Dysfunction in Early Diabetic Nephropathy |
|  | Proteins Involved in Diabetic Nephropathy |
|  | Mesangial Cell Dysfunction in Diabetic Nephropathy |
|  | Proteins Involved in Diabetic Neuropathy |
|  | Microangiopathy in Diabetic Neuropathy |
|  | Adipokines and Cytokines in Insulin Resistance in Skeletal Muscles |
|  | Proteins Involved in Insulin Resistance |
|  | Adipose Tissue Inflammation Provokes Insulin Resistance |
|  | Adiponectin Synthesis Declines in Insulin Resistance |
|  | Insulin Resistance in Hepatocytes |
|  | Hepatic Stellate Cells in non-Alcoholic Fatty Liver Disease |
|  | Proteins Involved in non-Alcoholic Fatty Liver Disease |
|  | Oxidation of Fatty Acid in non-Alcoholic Fatty Liver Disease |
|  | Thyroid Hormones in Adipose Tissue Metabolism |
|  | Proteins Involved in Graves Disease |
|  | Graves Ophthalmopathy |
|  | Thymic Follicular Hyperplasia |
|  | Proteins Involved in Myasthenia Gravis |
|  | Fatty Acids in Hypertension and Atherosclerosis Development |
|  | Adipokines Production by Adipocyte Impaired in Obesity |
|  | Proteins Involved in Obesity |
|  | Proteins Involved in Celiac Disease |
|  | CD4+T-Cell Response in Celiac Disease |
|  | Proteins with Altered Expression in Celiac Disease |
|  | Proteins Involved in Colorectal Neoplasms |
|  | Metastatic Colorectal Cancer |
|  | Crohn's Disease |
|  | B-Cell Activation in Crohn's Disease |
|  | Th17-Cell Activation in Crohn's Disease |
|  | Proteins Involved in Inflammatory Bowel Diseases |
|  | Dendritic Cell Dysfunction in Crohn's disease |
|  | Dendritic Cell Function in Ulcerative Colitis |
|  | Nicotine anti-Inflammatory Effect in Ulcerative Colitis |
|  | Proteins Involved in Ulcerative Colitis |
|  | Antiphospholipid Antibodies in Endothelial Cell |
|  | Proteins Involved in B-Cell Chronic Lymphocytic Leukemia |
|  | Diffuse Large-B-Cell Lymphoma |
|  | Diffuse Large-B-Cell Lymphoma ABC Subtype |
|  | Proteins Involved in Hodgkin Lymphoma |
|  | Mantle Cell Lymphoma |
|  | TNFR/NF-kB Classical Pathway in Multiple Myeloma Plasma Cells |
|  | IL6/IGF1/VEGFA Signaling in Multiple Myeloma |
|  | Multiple Myeloma |
|  | Proteins Involved in Multiple Myeloma |
|  | NOTCH Signaling in Multiple Myeloma |
|  | TNFR/NF-kB Alternative Pathway in Multiple Myeloma Plasma Cells |
|  | Antiphospholipid Antibodies and Endothelial Cell Interaction |
|  | Proteins Involved in Thrombophilia |
|  | SERPINA1 Associated Liver Damage |
|  | TRPM7/8 in Pancreatic Adenocarcinoma |
|  | Eosinophil Survival in Asthma |
|  | Airway Smooth Muscle Cell High Rate Proliferation |
|  | IgE Induces Airway Smooth Muscle Cell Proliferation |
|  | Basophil Activation in Asthma |
|  | Mast-Cell Related Chemokines Signaling in Asthma |
|  | Th17-Cell Differentiation in Asthma |
|  | Mucin Production in Goblet Airway Epithelial Cells |
|  | Airway Epithelial Cell Dysfunction |
|  | Proteins Involved in Gout |
|  | Osteoclast Activation in Gout |
|  | Neutrophil Recruitment in Sinovium in Gout |
|  | Monocytes Function in Gout |
|  | Proteins Involved in Osteoarthritis |
|  | Proteins Involved in Polycystic Ovary Syndrome |
|  | Dendritic Cell Dysfunction in Psoriatic Arthritis |
|  | Th17-Cell and Th1 Immune Responsein Psoriatic Arthritis |
|  | Osteoclast Activation in Psoriatic Arthritis |
|  | Synovial Fibroblast Activation in Psoriatic Arthritis |
|  | Keratinocyte Activation in Psoriatic Arthritis |
|  | Proteins Involved in Psoriatic Arthritis |
|  | Synovial Fibroblast Proliferation in Rheumatoid Arthritis |
|  | Osteoclast Activation in Rheumatoid Arthritis |
|  | TLR2 Induced Synovial Fibroblast Activation in Rheumatoid Arthritis |
|  | Proteins Involved in Rheumatoid Arthritis |
|  | B-Cells Function in Systemic Scleroderma |
|  | Genes with Mutations Associated with Systemic Scleroderma |
|  | Proteins Involved in Systemic Scleroderma |
|  | Skin Fibrosis |
|  | Proteins with Altered Expression in Systemic Scleroderma |
|  | Proteins Involved in HIV Type 1 Infection |
|  | Th17-Cell Numbers Reduction in HIV |
|  | CD4+ T-Cell Function Decline in HIV |
|  | Toll-like Receptors (TLR) in Periodontitis |
|  | Diabetes Induced Periodontitis |
|  | Estrogen Effects on Pregnancy Gingivitis |
|  | Proteins Involved in Periodontitis |
|  | PAPA Syndrome |
|  | TNF Receptor Associated Periodic Syndrome |
|  | Mevalonate Kinase Deficiency |
|  | IL36 Receptor Antagonist (IL1F5) Deficiency (DITRA) |
|  | Cryopyrin (NLPR3) Associated Periodic Syndromes |
|  | Familial Cold Autoinflammatory Syndrome 2 |
|  | Familial Mediterranean Fever |
|  | Majeed Syndrome |
|  | NF-kB Signaling Activation in Huntington Disease |
|  | Oxytocin Signaling in Brain Nerve Cell (Hypothesis) |
|  | Proteins Involved in Autistic Disorder |
|  | Drugs Targets in Autistic Disoreder (Hypothesis) |
|  | Proteins Involved in Autistic Disorder and Aggression |
|  | IL6 and Insulin Resistance in Muscular Dystrophies |
|  | IL6 Promotes Inflammation in Muscular Dystrophies |
|  | IL6 Overexpression After Contraction in Skeletal Muscles in Muscular Dystrophies |
|  | Proteins Involved in Osteoporosis |
|  | Bone Loss in Osteoporosis |
|  | Proteins with Altered Expression in Psoriatic Arthritis |
|  | Proteins with Altered Expression in Rheumatoid Arthritis |
|  | Microglia Activation in Alzheimer's Disease |
|  | Proteins Involved in Alzheimer's Disease |
|  | Proteins Involved in Epilepsy |
|  | Ketogenic Diet Mechanism (Hypothesis) |
|  | Blood-Brain Barrier Disruption in Epileptiform Disorders |
|  | HMGB1 and IL1B in Neuroinflammation |
|  | ADK Expression Downregulation after Acute Seizures |
|  | Proteins Involved in Glioblastoma |
|  | Proteins Involved in Astrocytoma |
|  | Proteins Involved in Glioma |
|  | Glioma Stem Cell Program Activation |
|  | Proteins Involved in Medulloblastoma |
|  | Proteins Involved in Migraine |
|  | Proteins Involved in Neuroblastoma |
|  | Microglia Activation in Parkinson's Disease |
|  | Proteins with Altered Expression in Parkinson's Disease |
|  | Proteins Involved in Parkinson's Disease |
|  | Proteins with Altered Expression in Amyotrophic Lateral Sclerosis |
|  | Proteins Involved in Amyotrophic Lateral Sclerosis |
|  | Microglia and Motor Neuron Interaction Dysregulation |
|  | Neuroinflammation in Amyotrophic Lateral Sclerosis |
|  | MBP/MOG/PLP in Immune System Activation |
|  | Proteins Involved in Multiple Sclerosis |
|  | IL6/IL12 Signaling Activates Immune System in Multiple Sclerosis |
|  | Proteins Involved in Sleep Disorders |
|  | Proteins Involved in Vascular Dementia |
|  | Proteins Involved in Endometriosis |
|  | Coagulation Factors and PAR1/2 Receptors (F2R/F2RL1) in Endometriosis |
|  | Proteins Involved in Spontaneous Abortion |
|  | Proteins Involved in Cholesteatoma |
|  | IL6/JAK/STAT3 Signaling in Cholesteatoma |
|  | Cholesteatoma |
|  | Proteins Involved in HPV Infection |
|  | HPV E6 and E7 Induced Disruption of TNFR/NF-kB Signaling in Keratinocytes |
|  | Androgen Receptor/FKBP5 Signaling |
|  | Prostate Cancer |
|  | Proteins Involved in Prostate Cancer |
|  | Proteins Involved in Age-Related Macular Degeneration |
|  | ER Stress in Macular Degeneration |
|  | Proteins Involved in Noise-Induced Hearing Loss |
|  | Cochlear Inflammation (Noise-Induced) |
|  | Proteins Involved in Otitis Media |
|  | Cytokines Trigger Otitis Media |
|  | Immune System Role in Otitis Media |
|  | Alveolar Macrophage Negative Regulation Declined in COPD |
|  | Alveolar Macrophages Dysfunction in COPD |
|  | Proteins Involved in Chronic Obstructive Pulmonary Disease |
|  | T-Cell Cytotoxic Activation |
|  | Alveolar Epithelial Cell Dysfunction in COPD |
|  | Proteins Involved in Pulmonary Emphysema |
|  | Proteins with Altered Expression in Cystic Fibrosis |
|  | Proteins Involved in Erectile Dysfunction |
|  | Proteins Involved in Glomerulonephritis |
|  | Proteins with Altered Expression in Glomerulonephritis |
|  | Endothelial Cell Dysfunction in Glomerulonephritis |
|  | Mesangial Cell Dysfunction in Glomerulonephritis |
|  | Proteins with Altered Expression in Pyelonephritis |
|  | Toll-like Receptors in Pyelonephritis |
|  | Endothelial Cell Dysfunction in Pyelonephritis |
|  | HMGB1 Expression Targets |
|  | TTR Expression Targets |
|  | S100B Expression Targets |
|  | AREG -> NFATC Expression Target |
|  | AREG -> CREB/CREBBP Expression Targets |
|  | AREG -> STAT Expression Targets |
|  | AREG -> AP-1 Expression Targets |
|  | AREG -> FOXO3A Expression Target |
|  | Noradrenaline/Gs Expression Targets |
|  | Dopamine/Gs Expression Targets |
|  | Serotonin/Gs Expression Targets |
|  | Noreadrenaline/Gq Expression Targets |
|  | Epinephrine/Gs Expression Targets |
|  | Serotonin/Gq Expression Targets |
|  | Epinephrine/Gq Expression Targets |
|  | Dopamine/Gi Expression Targets |
|  | BMP2/BMPR2 Expression Targets |
|  | BMP7/BMPR2/ACVR2 Expression Targets |
|  | Elevated Receptors -> Expression Targets in Joint |
|  | Elevated Receptors -> Expression Targets in Bone and Joint |
|  | CD2 Expression Targets |
|  | CD19 Expression Targets |
|  | CD19/CR2 Expression Targets |
|  | CD8 -> ATF/CREB/CREBBP Expression Targets |
|  | CD8 -> NF-kB Expression Targets |
|  | CD80 -> AP-1 Expression Targets |
|  | CD80 -> STAT Expression Targets |
|  | CD80 -> NF-kB Expression Targets |
|  | CD80 -> ATF/CREB/CREBBP Expression Targets |
|  | CD86 -> NFATC Expression Targets |
|  | CD86 -> ATF/CREB/CREBBP Expression Targets |
|  | CD86 -> STAT Expression Targets |
|  | CD86 -> NF-kB Expression Targets |
|  | CTF1 Expression Targets |
|  | CXCL2 Expression Targets |
|  | CXCL5 Expression Targets |
|  | CXCL12 Expression Targets |
|  | CCL3L1 Expression Target |
|  | CCL7 Expression Target |
|  | CCL15 Expression Targets |
|  | IL8 Expression Targets |
|  | CSF3 Expression Targets |
|  | CSF2 -> NF-kB Expression Targets |
|  | CSF2 -> STAT Expression Targets |
|  | LIF Expression Targets |
|  | EGF -> CTNN Expression Targets |
|  | EGF -> AP-1/ATF Expression Targets |
|  | EGF -> CREB/CREBBP/ELK/SRF/MYC Expression Targets |
|  | EGF -> MEF/MYOD/NFATC Expression Targets |
|  | EGF -> STAT Expression Targets |
|  | F2 -> AP-1/CREB/ELK/SRF/SP1 Expression Targets |
|  | F2 -> STAT1/NF-kB Expression Targets |
|  | FGF2 -> STAT Expression Targets |
|  | FGF18 -> STAT Expression Targets |
|  | FGF18 -> AP-1/CREB Expression Targets |
|  | FGF2 -> AP-1/CREB/CREBBP/ELK/SRF/MYC Expression Targets |
|  | GCG Expression Targets |
|  | FSHR Expression Targets |
|  | Glutamate/Gq Expression Targets |
|  | VIP Expression Targets |
|  | Elevated Receptors -> Expression Targets in Liver |
|  | Elevated Receptors -> Expression Targets in Mouth |
|  | GH1/GHR -> NF-kB/ELK/SRF/MYC Expression Targets |
|  | GH1/GHR -> STAT Expression Targets |
|  | GH1/PRLR Expression Targets |
|  | HGF -> STAT Expression Targets |
|  | HGF -> AP-1/CREB/ELK/SRF/MYC Expression Targets |
|  | TAM Receptor Signaling Activation in Cancer |
|  | Receptors and Adaptor Proteins Activated in Cancer |
|  | Proteins with Altered Expression in Cancer-Associated Sustaining of Proliferative Signaling |
|  | STAT3 and NFkB Activate Inflammation-Induced Tumorigenesis |
|  | IL23A and IL17A Provoke Cancer-Associated Inflammation |
|  | STAT3 Inhibits Maturation of Dendritic Cells and Facilitates Cancer Progression |
|  | Proteins with Altered Expression in Tumor-Promoting Inflammation |
|  | N2 Neutrophils in Tumor-Promoting Inflammation and Tumor Progression |
|  | VEGFA Dependent Angiogenesis in Cancer |
|  | Proteins with Altered Expression in Cancer Metastases |
|  | Epithelial to Mesenchymal Transition in Cancer: Overview |
|  | Cancer Cells Inhibit Adipocyte Differentiation |
|  | Skeletal Muscle Wasting in Cancer Cachexia |
|  | Tumor Infiltrating Macrophages in Cancer Progression and Immune Escape |
|  | Myeloid Derived Suppressor Cells in Cancer Immune Escape |
|  | IDO1 in Cancer Immune Escape |
|  | Proteins with Altered Expression in Cancer Immune Escape |
|  | IGF2 -> STAT Expression Targets |
|  | INHBA/ACVR2/BMPR Expression Targets |
|  | Insulin -> STAT Expression Targets |
|  | Insulin -> CEBPA/CTNNB/FOXA/FOXO Expression Targets |
|  | Insulin -> MEF/MYOD Expression Targets |
|  | Insulin -> ELK/SRF/HIF1A/MYC/SREBF Expression Targets |
|  | CTGF -> AP-1/CREB/MYC Expression Targets |
|  | Fibrinogen Expression Targets |
|  | IFNG/IFNR Expression Targets |
|  | IL6R -> CEBP/ELK/SRF Signaling |
|  | IL6R -> STAT Signaling |
|  | IL6ST -> STAT5B Signaling |
|  | IL7 Expression Targets |
|  | IL13 Expression Targets |
|  | IL21 Expression Targets |
|  | IL15 Expression Targets |
|  | IL16 -> AP-1 Expression Targets |
|  | IL3 Expression Targets |
|  | IL16 -> STAT Expression Targets |
|  | IL6 Expression Targets |
|  | IL5 Expression Targets |
|  | IL11 Expression Targets |
|  | IL16-> ATF/CREB/CREBBP Expression Target |
|  | IL2 Expression Targets |
|  | IL16 -> NF-kB Expression Targets |
|  | PTPRC -> BCL6 Expression Targets |
|  | Leptin -> STAT Expression Targets |
|  | Leptin -> ELK/SRF Expression Targets |
|  | Prostaglandin F Expression Targets |
|  | Thromboxane A2 Expression Targets |
|  | LPA Expression Targets |
|  | PAF/Gq -> AP-1/ATF1/CREB/ERK/SRF Expression Targets |
|  | Dronabinol/Anandamide Expression Targets |
|  | Morphine Expression Targets |
|  | S1P Expression Targets |
|  | PGE1 Expression Targets |
|  | Proteins/Ions Transporters -> Expression Targets in Lymphoid System and Blood |
|  | TGF-beta Receptors Family -> Expression Targets in Lymphoid System and Blood |
|  | Elevated Receptors -> Expression Targets in Lymph Node |
|  | Elevated Receptors -> Expression Targets in Lymphoid System and Blood |
|  | CD Markers -> Expression Targets in Lymphoid System and Blood |
|  | GPCRs Family -> Expression Targets in Lymphoid System and Blood |
|  | Cell Adhesion Receptors -> Expression Targets in Lymphoid System and Blood |
|  | TNF Receptors -> Expression Targets in Lymphoid System and Blood |
|  | Receptor Tyrosine Kinase Family -> Expression Targets in Lymphoid System and Blood |
|  | Interleukin Receptors -> Expression Targets in Lymphoid System and Blood |
|  | C5AR1 -> Expression Targets in Lymphoid System and Blood |
|  | IgE Receptors -> Targets in Lymphoid System and Blood |
|  | Elevated Receptors -> Expression Targets in Spleen |
|  | Elevated Receptors -> Expression Targets in Thymus |
|  | CNR1-> Expression Targets in Nerve Tissue |
|  | GPCRs -> Expression Targets in Brain |
|  | Elevated Receptors -> Expression Targets in Nerve Tissue |
|  | NGF -> SMAD3/NF-kB Expression Targets |
|  | NGF -> AP-1/TP53/MYC Expression Targets |
|  | NGF -> CREB/CEBPB/MEF2A Expression Targets |
|  | DLL4 Expression Targets |
|  | ADAM17 Expression Targets |
|  | NOTCH Expression Targets |
|  | Adenosine Expression Targets |
|  | OSM/OSMR Expression Targets |
|  | OSM/IL6ST/LIFR Expression Targets |
|  | PDGF -> AP-1/CREB/CREBBP/MYC Expression Targets |
|  | PDGF -> STAT Expression Targets |
|  | GNRH1 Expression Targets |
|  | EDN1 Expression Targets |
|  | AGT -> CREB Expression Targets |
|  | SST Expression Targets |
|  | IFNA1/Gq Expression Targets |
|  | TAC1 Expression Targets |
|  | EDN3 Expression Targets |
|  | AGT -> STAT Expression Targets |
|  | PRL/PRLR Expression Targets |
|  | PRL/GHR -> STAT Expression Targets |
|  | PRL/GHR -> NF/kB/ELK/SRF/MYC Expression Targets |
|  | CSF1 -> STAT Expression Targets |
|  | KITLG -> STAT Expression Targets |
|  | KITLG -> AP-1/CREB/CREBBP/MYC Expression Targets |
|  | Collagen -> NF-kB Expression Targets |
|  | PAF Expression Targets |
|  | CSF1 -> AP-1/CREB/CREBBP/MYC Expression Targets |
|  | PGF -> AP-1/CREB/CREBBP/MYC Expression Targets |
|  | MSTN/ACVR2/BMPR Expression Targets |
|  | Elevated Receptors -> Expression Targets in Lung |
|  | Elevated Receptors -> Expression Targets in Respiratory System |
|  | Estrogens/ESR1 Genomic Canonical Signaling |
|  | FOXO1 Signaling |
|  | MERTK Signaling |
|  | TCR -> CREB/CREBBP/ATF Expression Targets |
|  | TCR -> NF-kB Expression Targets |
|  | TCR -> STAT Expression Targets |
|  | TCR -> AP-1 Expression Targets |
|  | TGFB2-TGFBR1 Expression Targets |
|  | TGFB3-TGFBR1 Expression Targets |
|  | TGFB1-TGFBR1 Expression Targets |
|  | TGFB2-TGFBR2 Expression Targets |
|  | TGFB1-TGFBR2 Expression Targets |
|  | TGFB1-TGFBR1/AP-1 Expression Targets |
|  | TGFB1-ACVRL1 Expression Targets |
|  | TNF -> NF-kB Expression Targets |
|  | LTA Expression Targets |
|  | TNFSF14 Expression Targets |
|  | TNF -> AP-1 Expression Targets |
|  | CD72 -> AP-1 Expression Targets |
|  | CD40LG -> STAT Expression Targets |
|  | CD40LG -> NF-kB/ELK/SRF -> CREB/NFATC Expression Targets |
|  | TNF -> CREB Expression Targets |
|  | TNF -> STAT Expression Targets |
|  | TLR3 Expression Targets |
|  | TLR4 -> AP-1 Expression Targets |
|  | TLR5 Expression Targets |
|  | TLR7 Expression Targets |
|  | IL1B -> PGE2 Expression Targets |
|  | TLR1 -> 2/6 Expression Targets |
|  | IL1B Expression Targets |
|  | TLR4 -> NF-kB/IRF Expression Targets |
|  | IL1A Expression Targets |
|  | TLR9 Expression Targets |
|  | ROS in Triggering Vascular Inflammation |
|  | WNT5A Expression Targets |
|  | pathway 3 |
|  | PPAR Psoriasis |
|  | prarg negative regulators, ps-positive |
|  | pprarg neg, uknown targets, ps-positive |
|  | prarg neg,ukn expres targets, ps-positive |
|  | PPARG negative regulators |
|  | PPARG negative targets |
|  | pparg expr target\_regulators |
|  | Neighbors of disease exacerbation |
|  | Neighbors of tumor progression |
|  | Neighbors of cancer progression |
|  | Neighbors of inflammatory status |
|  | Neighbors of overall survival |
|  | Neighbors of disease severity |
|  | Neighbors of tumor size |
|  | Neighbors of lesion size |
|  | Neighbors of mortality |
|  | Neighbors of survival rate |
|  | Neighbors of infarct size |
|  | Neighbors of survival time |
|  | Neighbors of functional recovery |
|  | Neighbors of insulin sensitivity |
|  | Neighbors of bacterial load |
|  | Neighbors of cancer survival |
|  | Neighbors of graft survival |
|  | Neighbors of therapeutic efficacy |
|  | Neighbors of clinical stage |
|  | Neighbors of lethality |
|  | Neighbors of tumor regression |
|  | Neighbors of recurrence-free survival |
|  | Neighbors of disease-free survival |
|  | Neighbors of tumor response |
|  | Neighbors of macrophage count |
|  | Neighbors of long-term survival |
|  | Neighbors of treatment outcome |
|  | Neighbors of bone mass |
|  | Neighbors of body weight |
|  | Neighbors of body weight gain |
|  | Neighbors of cardiovascular risk |
|  | Neighbors of microvessel density |
|  | Neighbors of tumor recurrence |
|  | Neighbors of tumor promotion |
|  | Neighbors of neutrophil count |
|  | Neighbors of glucose tolerance |
|  | Neighbors of morbidity |
|  | Neighbors of fibrosis progression |
|  | Neighbors of mortality rate |
|  | Neighbors of fat mass |
|  | Neighbors of exhaustion |
|  | Neighbors of bone mineral density |
|  | Neighbors of adverse outcome |
|  | Neighbors of radioresistance |
|  | Neighbors of lesion area |
|  | Neighbors of cell density |
|  | Neighbors of cancer-specific survival |
|  | Neighbors of progression-free survival |
|  | Neighbors of apoptosis rate |
|  | Neighbors of early mortality |
|  | Neighbors of shear stress |
|  | Neighbors of viral load |
|  | Neighbors of blood pressure |
|  | Neighbors of leukocyte count |
|  | Neighbors of vascular reactivity |
|  | Neighbors of vascular leakage |
|  | Neighbors of body mass index |
|  | Neighbors of vascular density |
|  | Neighbors of muscle mass |
|  | Neighbors of cancer risk |
|  | Neighbors of cancer incidence |
|  | Neighbors of tumor aggressiveness |
|  | Neighbors of systolic blood pressure |
|  | Neighbors of tumor resistance |
|  | Neighbors of blood glucose |
|  | Neighbors of ejection fraction |
|  | Neighbors of median survival |
|  | Neighbors of renal hypertrophy |
|  | Neighbors of fasting plasma glucose |
|  | Neighbors of osteoclast number |
|  | Neighbors of cancer recurrence |
|  | Neighbors of T-cell count |
|  | Neighbors of energy expenditure |
|  | Neighbors of glucose level |
|  | Neighbors of vascular tone |
|  | Neighbors of radiosensitivity |
|  | Neighbors of left ventricular ejection fraction |
|  | Neighbors of breast cancer risk |
|  | Neighbors of cell permeability |
|  | Neighbors of pregnancy outcome |
|  | Neighbors of functional status |
|  | Neighbors of gastric cancer risk |
|  | Neighbors of neurological outcome |
|  | Neighbors of infarct area |
|  | Neighbors of fertility |
|  | Neighbors of recurrence rate |
|  | Neighbors of pathological response |
|  | Neighbors of lymphocyte count |
|  | Neighbors of monocyte count |
|  | Neighbors of skin thickness |
|  | Neighbors of metabolic response |
|  | Neighbors of insulin level |
|  | Neighbors of comorbidity |
|  | Neighbors of carotid intima-media thickness |
|  | Neighbors of bone volume |
|  | Neighbors of fibroblast phenotype |
|  | Neighbors of disease exacerbation |
|  | Neighbors of tumor progression |
|  | Neighbors of cancer progression |
|  | Neighbors of inflammatory status |
|  | Neighbors of overall survival |
|  | Neighbors of disease severity |
|  | Neighbors of tumor size |
|  | Neighbors of lesion size |
|  | Neighbors of mortality |
|  | Neighbors of survival rate |
|  | Neighbors of infarct size |
|  | Neighbors of survival time |
|  | Neighbors of functional recovery |
|  | Neighbors of insulin sensitivity |
|  | Neighbors of bacterial load |
|  | Neighbors of cancer survival |
|  | Neighbors of graft survival |
|  | Neighbors of therapeutic efficacy |
|  | Neighbors of clinical stage |
|  | Neighbors of lethality |
|  | Neighbors of tumor regression |
|  | Neighbors of recurrence-free survival |
|  | Neighbors of disease-free survival |
|  | Neighbors of tumor response |
|  | Neighbors of macrophage count |
|  | Neighbors of long-term survival |
|  | Neighbors of treatment outcome |
|  | Neighbors of bone mass |
|  | Neighbors of body weight |
|  | Neighbors of body weight gain |
|  | Neighbors of cardiovascular risk |
|  | Neighbors of microvessel density |
|  | Neighbors of tumor recurrence |
|  | Neighbors of tumor promotion |
|  | Neighbors of neutrophil count |
|  | Neighbors of glucose tolerance |
|  | Neighbors of morbidity |
|  | Neighbors of fibrosis progression |
|  | Neighbors of mortality rate |
|  | Neighbors of fat mass |
|  | Neighbors of exhaustion |
|  | Neighbors of bone mineral density |
|  | Neighbors of adverse outcome |
|  | Neighbors of radioresistance |
|  | Neighbors of lesion area |
|  | Neighbors of cell density |
|  | Neighbors of cancer-specific survival |
|  | Neighbors of progression-free survival |
|  | Neighbors of apoptosis rate |
|  | Neighbors of early mortality |
|  | Neighbors of shear stress |
|  | Neighbors of viral load |
|  | Neighbors of blood pressure |
|  | Neighbors of leukocyte count |
|  | Neighbors of vascular reactivity |
|  | Neighbors of vascular leakage |
|  | Neighbors of body mass index |
|  | Neighbors of vascular density |
|  | Neighbors of muscle mass |
|  | Neighbors of cancer risk |
|  | Neighbors of cancer incidence |
|  | Neighbors of tumor aggressiveness |
|  | Neighbors of systolic blood pressure |
|  | Neighbors of tumor resistance |
|  | Neighbors of blood glucose |
|  | Neighbors of ejection fraction |
|  | Neighbors of median survival |
|  | Neighbors of renal hypertrophy |
|  | Neighbors of fasting plasma glucose |
|  | Neighbors of osteoclast number |
|  | Neighbors of cancer recurrence |
|  | Neighbors of T-cell count |
|  | Neighbors of energy expenditure |
|  | Neighbors of glucose level |
|  | Neighbors of vascular tone |
|  | Neighbors of radiosensitivity |
|  | Neighbors of left ventricular ejection fraction |
|  | Neighbors of breast cancer risk |
|  | Neighbors of cell permeability |
|  | Neighbors of pregnancy outcome |
|  | Neighbors of functional status |
|  | Neighbors of gastric cancer risk |
|  | Neighbors of neurological outcome |
|  | Neighbors of infarct area |
|  | Neighbors of fertility |
|  | Neighbors of recurrence rate |
|  | Neighbors of pathological response |
|  | Neighbors of lymphocyte count |
|  | Neighbors of monocyte count |
|  | Neighbors of skin thickness |
|  | Neighbors of metabolic response |
|  | Neighbors of insulin level |
|  | Neighbors of comorbidity |
|  | Neighbors of carotid intima-media thickness |
|  | Neighbors of bone volume |
|  | Neighbors of fibroblast phenotype |
|  | Neighbors of cell development |
|  | Neighbors of immune response |
|  | Neighbors of cell population |
|  | Neighbors of T-cell development |
|  | Neighbors of inflammatory response |
|  | Neighbors of wound healing |
|  | Neighbors of angiogenesis |
|  | Neighbors of innate immune response |
|  | Neighbors of cell function |
|  | Neighbors of macrophage activation |
|  | Neighbors of cell formation |
|  | Neighbors of adaptive immune response |
|  | Neighbors of osteoclast development |
|  | Neighbors of epithelial to mesenchymal transition |
|  | Neighbors of immunity |
|  | Neighbors of cell proliferative response |
|  | Neighbors of chemotaxis |
|  | Neighbors of dendritic cell differentiation |
|  | Neighbors of cell survival |
|  | Neighbors of osteoclast differentiation |
|  | Neighbors of cell phenotype |
|  | Neighbors of T-cell proliferation |
|  | Neighbors of tissue remodeling |
|  | Neighbors of cell adhesion |
|  | Neighbors of T-cell activation |
|  | Neighbors of endothelial cell proliferation |
|  | Neighbors of fibrogenesis |
|  | Neighbors of tumor growth |
|  | Neighbors of dendritic cell development |
|  | Neighbors of tissue repair |
|  | Neighbors of cell count |
|  | Neighbors of cell infiltration |
|  | Neighbors of cell interaction |
|  | Neighbors of SMC proliferation |
|  | Neighbors of lumen formation |
|  | Neighbors of endothelial cell migration |
|  | Neighbors of adipogenesis |
|  | Neighbors of lipid storage |
|  | Neighbors of cell damage |
|  | Neighbors of pregnancy |
|  | Neighbors of monocyte differentiation |
|  | Neighbors of aging |
|  | Neighbors of cell motility |
|  | Neighbors of bone resorption |
|  | Neighbors of monocyte migration |
|  | Neighbors of fibroblast proliferation |
|  | Neighbors of adipocyte differentiation |
|  | Neighbors of macrophage migration |
|  | Neighbors of microglial activation |
|  | Neighbors of neutrophil recruitment |
|  | Neighbors of regeneration |
|  | Neighbors of cell migration |
|  | Neighbors of cancer growth |
|  | Neighbors of stem cell proliferation |
|  | Neighbors of smooth muscle cell migration |
|  | Neighbors of hepatic regeneration |
|  | Neighbors of transendothelial migration |
|  | Neighbors of phagocytosis |
|  | Neighbors of immune system activation |
|  | Neighbors of leukocyte migration |
|  | Neighbors of leukocyte recruitment |
|  | Neighbors of osteoclast formation |
|  | Neighbors of monocyte recruitment |
|  | Neighbors of cell invasion |
|  | Neighbors of heart function |
|  | Neighbors of hemopoiesis |
|  | Neighbors of bone remodeling |
|  | Neighbors of ossification |
|  | Neighbors of T-cell function |
|  | Neighbors of immune system function |
|  | Neighbors of lung development |
|  | Neighbors of autophagy |
|  | Neighbors of monocyte adhesion |
|  | Neighbors of transcription activation |
|  | Neighbors of senescence |
|  | Neighbors of T-cell response |
|  | Neighbors of tumor immunity |
|  | Neighbors of hepatocyte proliferation |
|  | Neighbors of cell transdifferentiation |
|  | Neighbors of neuroprotection |
|  | Neighbors of stem cell differentiation |
|  | Neighbors of endothelial cell function |
|  | Neighbors of neuronal death |
|  | Neighbors of cell growth |
|  | Neighbors of ROS generation |
|  | Neighbors of epithelial cell proliferation |
|  | Neighbors of first trimester pregnancy |
|  | Neighbors of cancer cell growth |
|  | Neighbors of sensitization |
|  | Neighbors of hemato-encephalic barrier |
|  | Neighbors of macrophage function |
|  | Neighbors of cellular immune response |
|  | Neighbors of cellular senescence |
|  | Neighbors of colony formation |
|  | Neighbors of macrophage differentiation |
|  | Neighbors of cell differentiation |
|  | Neighbors of keratinocyte proliferation |
|  | Neighbors of stem cell migration |
|  | Neighbors of life span |
|  | Neighbors of cell homeostasis |
|  | Neighbors of psoriasis |
|  | Neighbors of inflammatory disease |
|  | Neighbors of rheumatoid arthritis |
|  | Neighbors of atherosclerosis |
|  | Neighbors of fibrosis |
|  | Neighbors of colitis |
|  | Neighbors of arthritis |
|  | Neighbors of inflammatory bowel disease |
|  | Neighbors of injury |
|  | Neighbors of atherogenesis |
|  | Neighbors of vascular remodeling |
|  | Neighbors of metastasis |
|  | Neighbors of carcinogenesis |
|  | Neighbors of multiple sclerosis |
|  | Neighbors of chronic inflammation |
|  | Neighbors of inflammation |
|  | Neighbors of tumor microenvironment |
|  | Neighbors of pathological angiogenesis |
|  | Neighbors of obesity |
|  | Neighbors of vascular disease |
|  | Neighbors of diabetes mellitus |
|  | Neighbors of asthma |
|  | Neighbors of pneumonia |
|  | Neighbors of hyperplasia |
|  | Neighbors of osteoarthritis |
|  | Neighbors of liver fibrosis |
|  | Neighbors of chronic obstructive pulmonary disease |
|  | Neighbors of myocardial infarction |
|  | Neighbors of leukocyte infiltration |
|  | Neighbors of reperfusion injury |
|  | Neighbors of airway inflammation |
|  | Neighbors of hepatitis |
|  | Neighbors of insulin resistance |
|  | Neighbors of infection |
|  | Neighbors of vascular injury |
|  | Neighbors of renal injury |
|  | Neighbors of preeclampsia |
|  | Neighbors of macrophage infiltration |
|  | Neighbors of liver injury |
|  | Neighbors of neuroinflammation |
|  | Neighbors of sepsis |
|  | Neighbors of death |
|  | Neighbors of type 1 diabetes |
|  | Neighbors of encephalomyelitis |
|  | Neighbors of autoimmune disease |
|  | Neighbors of ischemic stroke |
|  | Neighbors of dermatitis |
|  | Neighbors of neutrophil infiltration |
|  | Neighbors of heart disease |
|  | Neighbors of diabetic nephropathy |
|  | Neighbors of lung injury |
|  | Neighbors of immunopathology |
|  | Neighbors of systemic sclerosis |
|  | Neighbors of hepatocellular carcinoma |
|  | Neighbors of autoimmunity |
|  | Neighbors of acute lung injury |
|  | Neighbors of gastric cancer |
|  | Neighbors of atherosclerotic plaque |
|  | Neighbors of experimental autoimmune encephalomyelitis |
|  | Neighbors of colorectal cancer |
|  | Neighbors of cardiac remodeling |
|  | Neighbors of breast cancer |
|  | Neighbors of ischemia |
|  | Neighbors of systemic lupus erythematosus |
|  | Neighbors of melanoma |
|  | Neighbors of collagen-induced arthritis |
|  | Neighbors of cartilage degeneration |
|  | Neighbors of interstitial fibrosis |
|  | Neighbors of neoplasm invasion |
|  | Neighbors of kidney disease |
|  | Neighbors of heart failure |
|  | Neighbors of lung disease |
|  | Neighbors of pulmonary fibrosis |
|  | Neighbors of cardiovascular disease |
|  | Neighbors of stroke |
|  | Neighbors of cancer |
|  | Neighbors of cerebral ischemia |
|  | Neighbors of type 2 diabetes |
|  | Neighbors of neoplasm |
|  | Neighbors of endometriosis |
|  | Neighbors of neointima |
|  | Neighbors of vasculitis |
|  | Neighbors of atrophy |
|  | Neighbors of diet-induced obesity |
|  | Neighbors of respiratory hypersensitivity |
|  | Neighbors of myocardial fibrosis |
|  | Neighbors of synovitis |
|  | Neighbors of coronary artery disease |
|  | Neighbors of rupture |
|  | Neighbors of endothelial cell dysfunction |
|  | Neighbors of liver disease |
|  | Neighbors of prostate cancer |
|  | Neighbors of hypertrophy |
|  | Neighbors of osteolysis |
|  | Neighbors of ovarian cancer |
|  | Neighbors of pancreatic cancer |
|  | Neighbors of hypertension |
|  | Neighbors of intimal hyperplasia |
|  | Neighbors of allograft rejection |
|  | Neighbors of proteinuria |
|  | Neighbors of epithelial cell |
|  | Neighbors of keratinocyte |
|  | Neighbors of vascular smooth muscle cell |
|  | Neighbors of umbilical vein endothelial cell |
|  | Neighbors of monocyte |
|  | Neighbors of endometrial stromal cell |
|  | Neighbors of alveolar macrophage |
|  | Neighbors of synoviocyte |
|  | Neighbors of macrophage |
|  | Neighbors of adipocyte |
|  | Neighbors of activated monocyte |
|  | Neighbors of fibroblast |
|  | Neighbors of airway epithelial cell |
|  | Neighbors of PBMC |
|  | Neighbors of stromal cell |
|  | Neighbors of airway smooth muscle cell |
|  | Neighbors of osteoblast |
|  | Neighbors of pneumocyte |
|  | Neighbors of endometrium cell |
|  | Neighbors of microglia |
|  | Neighbors of trophoblast |
|  | Neighbors of intestine epithelium cell |
|  | Neighbors of mononuclear cell |
|  | Neighbors of hepatic stellate cell |
|  | Neighbors of hepatocyte |
|  | Neighbors of decidual cell |
|  | Neighbors of kidney mesangial cell |
|  | Neighbors of endothelial cell |
|  | Neighbors of dendritic cell |
|  | Neighbors of Kuppfer cell |
|  | Neighbors of proadipocyte |
|  | Neighbors of kidney tubule cell |
|  | Neighbors of inflammatory cell |
|  | Neighbors of bone marrow macrophage |
|  | Neighbors of peritoneal macrophage |
|  | Neighbors of osteoclast |
|  | Neighbors of neutrophil |
|  | Neighbors of smooth muscle myocyte |
|  | Neighbors of astrocyte |
|  | Neighbors of bone marrow derived dendritic cell |
|  | Neighbors of lung fibroblast |
|  | Neighbors of activated T-cell |
|  | Neighbors of mesothelial cell |
|  | Neighbors of mononuclear phagocyte |
|  | Neighbors of capillary endothelial cell |
|  | Neighbors of immunocompetent cell |
|  | Neighbors of granulocyte |
|  | Neighbors of cardiac myocyte |
|  | Neighbors of cancer-associated fibroblast |
|  | Neighbors of myeloid cell |
|  | Neighbors of mesenchymal stroma cell |
|  | Neighbors of corneal epithelial cell |
|  | Neighbors of cytotrophoblast |
|  | Neighbors of stem cell |
|  | Neighbors of tumor infiltrating macrophage |
|  | Neighbors of sinusoidal cell |
|  | Neighbors of B-cell |
|  | Neighbors of granulosa cell |
|  | Neighbors of leukocyte |
|  | Neighbors of chondrocyte |
|  | Neighbors of insulin-secreting cell |
|  | Neighbors of mesenchymal cell |
|  | Neighbors of T-cell |
|  | Neighbors of M1 macrophage |
|  | Neighbors of myofibroblast |
|  | Neighbors of pancreatic stellate cell |
|  | Neighbors of mast cell |
|  | Neighbors of bone marrow stromal cell |
|  | Neighbors of spleen cell |
|  | Neighbors of brain capillary endothelial cell |
|  | Neighbors of neuroglia |
|  | Neighbors of thyroid epithelial cell |
|  | Neighbors of aortic smooth muscle cell |
|  | Neighbors of hematopoietic cell |
|  | Neighbors of adipose macrophage |
|  | Neighbors of podocyte |
|  | Neighbors of mammary epithelial cell |
|  | Neighbors of conventional dendritic cell |
|  | Neighbors of pericyte |
|  | Neighbors of antigen-presenting cell |
|  | Neighbors of epidermis cell |
|  | Neighbors of bone marrow derived mast cell |
|  | Neighbors of phagocyte |
|  | Neighbors of kidney cell |
|  | Neighbors of syncytiotrophoblast |
|  | Neighbors of lymphocyte |
|  | Neighbors of Langerhans cell |
|  | Neighbors of decidual natural killer cell |
|  | Neighbors of thymic epithelial cell |
|  | Neighbors of brain cell |
|  | Neighbors of M2 macrophage |
|  | Neighbors of peritoneum cell |
|  | Neighbors of lymphoid cell |
|  | Neighbors of retina cell |
|  | Neighbors of blood cell |
|  | Neighbors of skin cell |
|  | Neighbors of luteal cell |
|  | Neighbors of peripheral lymphocyte |
|  | Neighbors of activated B-cell |
|  | Neighbors of lymph node cell |
|  | Neighbors of lung |
|  | Neighbors of liver |
|  | Neighbors of kidney |
|  | Neighbors of skin |
|  | Neighbors of intestine |
|  | Neighbors of lymph node |
|  | Neighbors of colon |
|  | Neighbors of airway |
|  | Neighbors of blood vessel |
|  | Neighbors of heart |
|  | Neighbors of aorta |
|  | Neighbors of gastrointestinal tract |
|  | Neighbors of microvessel |
|  | Neighbors of bone |
|  | Neighbors of placenta |
|  | Neighbors of carotid artery |
|  | Neighbors of synovial membrane |
|  | Neighbors of uterus |
|  | Neighbors of esophagus |
|  | Neighbors of periodontium |
|  | Neighbors of retina |
|  | Neighbors of brain |
|  | Neighbors of ovary |
|  | Neighbors of breast |
|  | Neighbors of blood vessel wall |
|  | Neighbors of stomach |
|  | Neighbors of islets of Langerhans |
|  | Neighbors of joint |
|  | Neighbors of artery |
|  | Neighbors of cardiovascular system |
|  | Neighbors of pancreas |
|  | Neighbors of prostate |
|  | Neighbors of central nervous system |
|  | Neighbors of kidney glomerulus |
|  | Neighbors of lymphatic system |
|  | Neighbors of colorectal region |
|  | Neighbors of decidua |
|  | Neighbors of amnion |
|  | Neighbors of immune system |
|  | Neighbors of spleen |
|  | Neighbors of peritoneum |
|  | Neighbors of allantois |
|  | Neighbors of alveolus |
|  | Neighbors of coronary artery |
|  | Neighbors of left ventricle |
|  | Neighbors of uterine cervix |
|  | Neighbors of omentum |
|  | Neighbors of vein |
|  | Neighbors of arteriole |
|  | Neighbors of villus |
|  | Neighbors of bronchus |
|  | Neighbors of glands |
|  | Neighbors of nose |
|  | Neighbors of eye |
|  | Neighbors of cornea |
|  | Neighbors of hippocampus |
|  | Neighbors of intervertebral disc |
|  | Neighbors of lymph node of mesentery |
|  | Neighbors of mammary gland |
|  | Neighbors of vagina |
|  | Neighbors of ankle joint |
|  | Neighbors of vitreous body |
|  | Neighbors of thymus gland |
|  | Neighbors of cerebral cortex |
|  | Neighbors of brain blood vessel |
|  | Neighbors of myelin sheath |
|  | Neighbors of bladder |
|  | Neighbors of draining lymph node |
|  | Neighbors of nervous system |
|  | Neighbors of blastocyst |
|  | Neighbors of retina blood vessel |
|  | Neighbors of periodontal ligament |
|  | Neighbors of salivary gland |
|  | Neighbors of trachea |
|  | Neighbors of corpus luteum of ovary |
|  | Neighbors of sympathetic nervous system |
|  | Neighbors of nerve |
|  | Neighbors of ileum |
|  | Neighbors of endocrine system |
|  | Neighbors of lower extremity |
|  | Neighbors of small intestine |
|  | Neighbors of aggregated lymphoid follicle of small intestine |
|  | Neighbors of globus pallidus |
|  | Neighbors of amniotic cavity |
|  | Neighbors of lumbar spine |
|  | Neighbors of knee joint |
|  | Neighbors of hip region |
|  | Neighbors of reproductive system |
|  | Neighbors of pleura |
|  | Neighbors of left atrium |
|  | Neighbors of hypothalamus |
|  | Neighbors of artery wall |
|  | Neighbors of NF-kB family |
|  | Neighbors of oxidized LDL |
|  | Neighbors of JNK |
|  | Neighbors of IL1 family |
|  | Neighbors of PPAR |
|  | Neighbors of Jun/Fos |
|  | Neighbors of NFKBI |
|  | Neighbors of PKC |
|  | Neighbors of PI3K |
|  | Neighbors of STAT family |
|  | Neighbors of mitogen-activated protein kinase |
|  | Neighbors of IFNAR ligand |
|  | Neighbors of transforming growth factor |
|  | Neighbors of TLR |
|  | Neighbors of cytokine |
|  | Neighbors of interferon |
|  | Neighbors of interleukin |
|  | Neighbors of JAK |
|  | Neighbors of MEK1/2 |
|  | Neighbors of PDGF |
|  | Neighbors of Ras GTPase |
|  | Neighbors of ERK1/2 |
|  | Neighbors of growth factor |
|  | Neighbors of PKA |
|  | Neighbors of ovalbumin |
|  | Neighbors of LDL |
|  | Neighbors of NF-AT family |
|  | Neighbors of beta adrenoceptor |
|  | Neighbors of chorionic gonadotropin |
|  | Neighbors of IKBK |
|  | Neighbors of adipokine |
|  | Neighbors of protein tyrosine kinase |
|  | Neighbors of NAD(P)H oxidase |
|  | Neighbors of IgG |
|  | Neighbors of SMAD subfamily |
|  | Neighbors of AMPK |
|  | Neighbors of LXR |
|  | Neighbors of HDL |
|  | Neighbors of p38 |
|  | Neighbors of inflammatory cytokine |
|  | Neighbors of IL23 |
|  | Neighbors of estrogen receptor |
|  | Neighbors of T-cell receptor |
|  | Neighbors of histone deacetylase |
|  | Neighbors of PDGF-BB |
|  | Neighbors of IL1R |
|  | Neighbors of HIF-1 |
|  | Neighbors of GPCR |
|  | Neighbors of IL12 |
|  | Neighbors of Notch |
|  | Neighbors of prostaglandin-endoperoxide synthase |
|  | Neighbors of heat shock protein 90 |
|  | Neighbors of lectin |
|  | Neighbors of fibrinogen |
|  | Neighbors of aldehyde reductase |
|  | Neighbors of mitogen-activated protein kinase kinase |
|  | Neighbors of interleukin-35 |
|  | Neighbors of TSH |
|  | Neighbors of Ang II receptor |
|  | Neighbors of endothelin |
|  | Neighbors of NOS |
|  | Neighbors of superoxide dismutase |
|  | Neighbors of proteasome endopeptidase complex |
|  | Neighbors of class nuclear receptor with C4 zinc fingers |
|  | Neighbors of non-coding RNA |
|  | Neighbors of histone H3 |
|  | Neighbors of STAT5 |
|  | Neighbors of alpha2 adrenoceptor |
|  | Neighbors of vitronectin receptor (integrin) |
|  | Neighbors of PP2A |
|  | Neighbors of retinoid-X receptor subfamily |
|  | Neighbors of SAPK |
|  | Neighbors of WNT |
|  | Neighbors of gonadotropins |
|  | Neighbors of PP2B |
|  | Neighbors of phospholipase |
|  | Neighbors of AHR subfamily |
|  | Neighbors of ITG |
|  | Neighbors of endostatins |
|  | Neighbors of CD3 complex |
|  | Neighbors of relaxin |
|  | Neighbors of TLR1/2 heterodimer |
|  | Neighbors of FSH |
|  | Neighbors of TORC1 |
|  | Neighbors of angiotensin receptor |
|  | Neighbors of RA receptor |
|  | Neighbors of histone |
|  | Neighbors of Rho kinase |
|  | Neighbors of PDGF receptor |
|  | Neighbors of neuropeptides |
|  | Neighbors of pancreatic elastase |
|  | Neighbors of histone acetyltransferase |
|  | Neighbors of adrenoceptor |
|  | Neighbors of phosphodiesterase |
|  | Neighbors of caspase |
|  | Neighbors of phosphoinositide phospholipase C |
|  | Neighbors of deacetylase |
|  | Neighbors of TLR4/Myd88 |
|  | Model of PPARG signaling in psoriasis |
|  | PPARG negative regulators and targets |
|  | Model of PPARG related pathways in psoriasis (short version) |
|  | PPARs and anxiety common targets |
|  | Amygdaloid expressed genes |
|  | PPARs and neuroinflamation |
|  | sars-cov no binding |
|  | dsRNA induces innate immunity and INFs in alveolar cells |
|  | IFN signaling in alveolar cells |
|  | Dendritic Cell Activation in COVID19 |
|  | Overview. Diseases of circulatory system (chapter 8) |
|  | Overview. Diseases of the skin (chapter 11) |
|  | Overview. Diseases of the digestive system (chapter 10) |
|  | Overview. Diseases of the musculoskeletal system (chapter 12) |
|  | Overview. Diseases of the nervous system (chapter 5) |
|  | Overview. Diseases of the respiratory (chapter 9) |
|  | Overview. Diseases of the genitourinary system (chapter 13) |
|  | Overview. Endocrine and metabolic diseases (chapter 4) |
|  | Overview. Infectious and parasitic diseases (chapter 2) |
|  | 1\_Differentiation of psoriatic T cells |
|  | 1\_The role of Toll-like receptors in pyelonephritis |
|  | 2\_The role of endothelial cells in pyelonephritis |
|  | 3\_Endoplasmic reticulum stress in age-related macular degeneration |
|  | 1\_Insufficient activation of immune response in the middle ear epithelium cells in otitis media |
|  | 2\_Cardiomyocyte hypertrophy |
|  | 1\_Cigarette smoke causes dysfunction and production of inflammatory mediators in alveolar epithelial cells |
|  | 3\_Role of alveolar macrophages in pulmonary tissue destruction in COPD |
|  | 1\_Th2 cell response in asthma |
|  | 3\_1\_Mucus accumulation in asthma |
|  | 2\_2\_Eosinophilia and neutrophilia in asthma: eosinophil apoptosis |
|  | 4\_Mucin hyperproduction in goblet and mucous cells in COPD |
|  | 1\_Defects in response to pathogens in the gut promotes inflammation in Crohn's disease |
|  | 2\_Intestine epithelial cells drive immunological responses in ulcerative colitis |
|  | 4\_1\_Polymorphisms associated with inflammatory bowel diseases |
|  | 2\_Lipotoxicity in NAFLD |
|  | 1\_CD4+ T cell and epithelial cell response in celiac disease |
|  | 6\_p12I Viral protein promotes T-cell activation in HTLV-1 infection |
|  | 5\_Human papilloma virus E6 and E7 proteins promote epithelial cell proliferation |
|  | 3\_Impairment of iron release from splenic macrophages |
|  | 1\_Inflammatory mediators increase the production of hepcidin (HAMP) |
|  | 4\_Inflammatory mediators inhibit GATA signaling in erythropoiesis |
|  | 4\_2\_Insulin resistance: inflammation-related insulin resistance |
|  | 4\_3\_Insulin resistance: FFA-related insulin resistance |
|  | 2\_SERPINA1-associated liver damage |
|  | 1\_Microangiopathy in diabetic neuropathy |
|  | drug-target pairs direct regulation |
|  | drug-target moltransport |
|  | New Combined Pathway |
|  | Differentiation of psoriatic T cells |

---

|  |  |
| --- | --- |
| Group | Genes with Mutations Associated with Atherosclerosis |
|  | Genes Associated with Systemic Lupus Erythematosus |
|  | Genes with Mutations Associated with Hashimoto's Thyroiditis |
|  | Proteins Involved in Graves Ophthalmopathy |
|  | Genes with Mutations Associated with Systemic Scleroderma |
|  | Genes with Mutations Associated with Multiple Sclerosis |
|  | Genes with Mutations Associated with Endometriosis |
|  | other partners for SARS |

---

|  |  |
| --- | --- |
| Source | curated |
|  | ResNet |
|  | GO component |

---

|  |  |
| --- | --- |
| MedScan ID | 3569 |

---

|  |  |
| --- | --- |
| LocusLink ID | 3569 |
|  | 16193 |
|  | 24498 |
|  | 280826 |

---

|  |  |
| --- | --- |
| Alias | interleukin IL6\_HUMAN |
|  | beta 2 interferon |
|  | BSF2 |
|  | IFNB2 |
|  | OTTHUMP00000158544 |
|  | OTTHUMP00000198486 |
|  | interleukin-6 receptor ligands |
|  | plasmacytoma growth factor |
|  | interferon beta-2 |
|  | IL-6 receptor ligand |
|  | interleukin BSF-2 |
|  | interferon beta II |
|  | interleukin-6 receptor ligand |
|  | B-cell stimulatory factor 2 |
|  | interleukin VI |
|  | IL6\_HUMAN |
|  | B lymphocyte stimulating factor 2 |
|  | interleukin IL6 |
|  | liver cell stimulating factor |
|  | IFN-beta-2 |
|  | beta interferon 2 |
|  | B-cell hybridoma growth factor |
|  | hepatocyte stimulating factor |
|  | IL6 |
|  | OTTHUMP00000198490 |
|  | ILg6 |
|  | hybridoma growth factor |
|  | interleukin 6 |
|  | CTL differentiation factor |
|  | IL6R ligand |
|  | interleukin b |
|  | B-cell stimulatory factor II |
|  | B-cell stimulating factor 2 |
|  | interleukin HP-1 |
|  | CDF |
|  | HGF |
|  | HSF |
|  | IL-6 |
|  | BSF-2 |
|  | B-cell differentiation factor |
|  | interleukin-6 |
|  | Interleukin 6 (interferon, beta 2) |
|  | interlukin 6 |
|  | interferon, beta 2 |
|  | B cell stimulatory factor-2 |
|  | Interleukin-IL6 |
|  | IL[6] |
|  | interleukin 6 (interferon |
|  | Interleukin 6 (interferon beta 2) |
|  | interleukin-6 precursor |

---

|  |  |
| --- | --- |
| GO ID | 0005125 |
|  | 0008083 |
|  | 0005138 |
|  | 0072540 |
|  | 0006953 |
|  | 0044267 |
|  | 0070301 |
|  | 0071222 |
|  | 0019221 |
|  | 0050829 |
|  | 0050830 |
|  | 0051607 |
|  | 0031018 |
|  | 0070091 |
|  | 0002384 |
|  | 0006959 |
|  | 0006954 |
|  | 0070102 |
|  | 0002548 |
|  | 0043066 |
|  | 0045779 |
|  | 0008285 |
|  | 0045079 |
|  | 0032966 |
|  | 0045599 |
|  | 2000660 |
|  | 0010888 |
|  | 0050768 |
|  | 0070050 |
|  | 0031175 |
|  | 0001781 |
|  | 0002446 |
|  | 0030168 |
|  | 0050871 |
|  | 0051091 |
|  | 0046427 |
|  | 0043410 |
|  | 0051092 |
|  | 0042102 |
|  | 2000553 |
|  | 0002675 |
|  | 1902512 |
|  | 0043065 |
|  | 0008284 |
|  | 0032722 |
|  | 0010718 |
|  | 0090091 |
|  | 0010628 |
|  | 0060252 |
|  | 0051024 |
|  | 0045380 |
|  | 0032755 |
|  | 0002690 |
|  | 0150078 |
|  | 0045669 |
|  | 0033138 |
|  | 0050731 |
|  | 0048661 |
|  | 0045944 |
|  | 0045893 |
|  | 0045727 |
|  | 2000676 |
|  | 0042531 |
|  | 0010575 |
|  | 0043687 |
|  | 0045765 |
|  | 0061888 |
|  | 1905603 |
|  | 1903978 |
|  | 0150077 |
|  | 0010574 |
|  | 0051384 |
|  | 0032494 |
|  | 0005788 |
|  | 0005576 |
|  | 0005615 |
|  | 0005896 |
|  | 0005102 |
|  | 0042110 |
|  | 0060445 |
|  | 0045454 |
|  | 0035729 |
|  | 0071347 |
|  | 0071356 |
|  | 0042832 |
|  | 0060664 |
|  | 0042593 |
|  | 0006955 |
|  | 0046716 |
|  | 0002262 |
|  | 0043154 |
|  | 0050710 |
|  | 0045721 |
|  | 0046888 |
|  | 0045837 |
|  | 0048635 |
|  | 1901215 |
|  | 0006469 |
|  | 0045740 |
|  | 0070374 |
|  | 0045630 |
|  | 0071864 |
|  | 0050679 |
|  | 0014015 |
|  | 0045666 |
|  | 0010976 |
|  | 0045429 |
|  | 0051897 |
|  | 0051971 |
|  | 0042981 |
|  | 0042127 |
|  | 0008360 |
|  | 0045188 |
|  | 0009611 |
|  | 0005737 |
|  | 0009897 |
|  | 0002526 |
|  | 0007568 |
|  | 0046849 |
|  | 0071345 |
|  | 0071549 |
|  | 0071392 |
|  | 0031669 |
|  | 1990646 |
|  | 0031294 |
|  | 2000866 |
|  | 0010469 |
|  | 0043200 |
|  | 0046677 |
|  | 0010996 |
|  | 0009617 |
|  | 0031000 |
|  | 0051592 |
|  | 0009409 |
|  | 0034097 |
|  | 0042493 |
|  | 0051602 |
|  | 0009408 |
|  | 0032868 |
|  | 0032496 |
|  | 0009612 |
|  | 0031667 |
|  | 0014070 |
|  | 0001878 |
|  | 0042060 |
|  | 0033160 |
|  | 0016049 |
|  | 2000366 |
|  | 0042517 |
|  | 0005623 |
|  | 0045768 |
|  | 0043526 |
|  | 0043193 |
|  | 0051789 |
|  | 0030072 |
|  | 0005515 |
|  | 0007435 |
|  | 0007166 |
|  | 0007267 |
|  | 0001501 |
|  | 0007275 |
|  | 0008283 |

---

|  |  |
| --- | --- |
| KEGG ID | hsa:3569 |
|  | mmu:16193 |
|  | rno:24498 |

---

|  |  |
| --- | --- |
| MedLine Reference | 11072134 |
|  | 11710966 |
|  | 11781188 |
|  | 11834488 |
|  | 11884027 |
|  | 11886848 |
|  | 11948286 |
|  | 12046086 |
|  | 12100026 |
|  | 12110437 |
|  | 12165795 |
|  | 12185323 |
|  | 12193539 |
|  | 12392281 |
|  | 12397370 |
|  | 12423624 |
|  | 12604335 |
|  | 12639960 |
|  | 12646246 |
|  | 12661899 |
|  | 12667648 |
|  | 12785009 |
|  | 12821383 |
|  | 12824010 |
|  | 12865171 |
|  | 12950323 |
|  | 12957790 |
|  | 12960020 |
|  | 14575314 |
|  | 14606087 |
|  | 14654230 |
|  | 14688199 |
|  | 14715528 |
|  | 14754894 |
|  | 15019946 |
|  | 15031602 |
|  | 15130702 |
|  | 15256805 |
|  | 15272006 |
|  | 15298985 |
|  | 15316242 |
|  | 15316244 |
|  | 15328477 |
|  | 15342195 |
|  | 15345694 |
|  | 15385469 |
|  | 15475534 |
|  | 15551000 |
|  | 15575898 |
|  | 15781751 |
|  | 15905211 |
|  | 15920760 |
|  | 16033193 |
|  | 16150725 |
|  | 16189509 |
|  | 16272873 |
|  | 16381797 |
|  | 16406266 |
|  | 16413512 |
|  | 16427158 |
|  | 16484300 |
|  | 16809219 |
|  | 16809483 |
|  | 16815050 |
|  | 16877563 |
|  | 16888221 |
|  | 16940225 |
|  | 17009398 |
|  | 17050174 |
|  | 17133487 |
|  | 2789217 |
|  | 8698878 |
|  | 10028917 |
|  | 10215902 |
|  | 10229817 |
|  | 10341234 |
|  | 10349636 |
|  | 10383128 |
|  | 10383613 |
|  | 10397473 |
|  | 10415153 |
|  | 10438933 |
|  | 10447717 |
|  | 10452974 |
|  | 10465257 |
|  | 10482564 |
|  | 10496318 |
|  | 10521784 |
|  | 10534116 |
|  | 10566878 |
|  | 10568881 |
|  | 10613740 |
|  | 10623431 |
|  | 10648128 |
|  | 10651996 |
|  | 10684643 |
|  | 10698075 |
|  | 10712626 |
|  | 10733552 |
|  | 10793074 |
|  | 10843805 |
|  | 10845903 |
|  | 10899153 |
|  | 10943842 |
|  | 10945466 |
|  | 10979125 |
|  | 11006436 |
|  | 11018077 |
|  | 11034996 |
|  | 11042159 |
|  | 11045959 |
|  | 11073809 |
|  | 11076861 |
|  | 11091275 |
|  | 11099471 |
|  | 11106580 |
|  | 11114976 |
|  | 11120777 |
|  | 11208530 |
|  | 11217851 |
|  | 11241200 |
|  | 11275690 |
|  | 11277977 |
|  | 11369703 |
|  | 11395027 |
|  | 11416016 |
|  | 11441074 |
|  | 11477542 |
|  | 11500099 |
|  | 11507020 |
|  | 11536174 |
|  | 11553589 |
|  | 11602623 |
|  | 11698278 |
|  | 11710994 |
|  | 11770048 |
|  | 11781184 |
|  | 11781288 |
|  | 11781360 |
|  | 11784794 |
|  | 11786910 |
|  | 11801681 |
|  | 11803046 |
|  | 11805288 |
|  | 11812493 |
|  | 11829488 |
|  | 11847480 |
|  | 11869683 |
|  | 11883745 |
|  | 11884029 |
|  | 11884396 |
|  | 11895333 |
|  | 11923871 |
|  | 11994291 |
|  | 12054913 |
|  | 12060750 |
|  | 12070012 |
|  | 12077263 |
|  | 12097416 |
|  | 12126646 |
|  | 12150983 |
|  | 12161103 |
|  | 12171912 |
|  | 12177065 |
|  | 12209330 |
|  | 12219085 |
|  | 12223557 |
|  | 12358740 |
|  | 12370360 |
|  | 12393639 |
|  | 12438448 |
|  | 12446574 |
|  | 12456808 |
|  | 12466851 |
|  | 12496437 |
|  | 12505667 |
|  | 12509437 |
|  | 12519742 |
|  | 12532024 |
|  | 12533608 |
|  | 12538435 |
|  | 12557750 |
|  | 12560330 |
|  | 12594267 |
|  | 12643284 |
|  | 12648490 |
|  | 12665506 |
|  | 12668153 |
|  | 12676746 |
|  | 12679717 |
|  | 12697697 |
|  | 12705907 |
|  | 12707321 |
|  | 12754505 |
|  | 12754507 |
|  | 12759566 |
|  | 12773301 |
|  | 12773503 |
|  | 12775331 |
|  | 12781987 |
|  | 12829028 |
|  | 12842862 |
|  | 12850812 |
|  | 12917264 |
|  | 12925700 |
|  | 12939594 |
|  | 12946594 |
|  | 12952969 |
|  | 12957371 |
|  | 12957791 |
|  | 12958147 |
|  | 12960349 |
|  | 12964032 |
|  | 1318227 |
|  | 1324224 |
|  | 1350248 |
|  | 1371079 |
|  | 1373998 |
|  | 1375756 |
|  | 1382446 |
|  | 1402392 |
|  | 14500666 |
|  | 14568951 |
|  | 14573621 |
|  | 14578297 |
|  | 14583241 |
|  | 14632648 |
|  | 14634061 |
|  | 14635056 |
|  | 14645573 |
|  | 14659593 |
|  | 14691481 |
|  | 14699015 |
|  | 14701687 |
|  | 14707102 |
|  | 1472283 |
|  | 14741447 |
|  | 14755335 |
|  | 14760941 |
|  | 14764608 |
|  | 14767066 |
|  | 14966572 |
|  | 14976041 |
|  | 14985352 |
|  | 14988384 |
|  | 14999682 |
|  | 15001458 |
|  | 15044588 |
|  | 15064729 |
|  | 15099921 |
|  | 15100312 |
|  | 15128857 |
|  | 15155665 |
|  | 15159136 |
|  | 15162832 |
|  | 15187118 |
|  | 15188379 |
|  | 15191552 |
|  | 15191885 |
|  | 15192150 |
|  | 15195698 |
|  | 15219849 |
|  | 15221959 |
|  | 15240714 |
|  | 15254764 |
|  | 15265638 |
|  | 15271886 |
|  | 15289466 |
|  | 15292196 |
|  | 15313196 |
|  | 15314695 |
|  | 15315756 |
|  | 15339938 |
|  | 15356132 |
|  | 15358102 |
|  | 15358232 |
|  | 15367390 |
|  | 15467358 |
|  | 15475383 |
|  | 15501797 |
|  | 15501801 |
|  | 15514851 |
|  | 15516495 |
|  | 15519344 |
|  | 15520045 |
|  | 1552482 |
|  | 15546395 |
|  | 15557373 |
|  | 15558055 |
|  | 15558059 |
|  | 15570035 |
|  | 15581736 |
|  | 15581737 |
|  | 15585847 |
|  | 15588510 |
|  | 15588696 |
|  | 15613028 |
|  | 15639639 |
|  | 15647834 |
|  | 15664785 |
|  | 15668925 |
|  | 15681696 |
|  | 15684062 |
|  | 15688401 |
|  | 15717324 |
|  | 15728480 |
|  | 15743795 |
|  | 15761498 |
|  | 15763341 |
|  | 15763541 |
|  | 1577194 |
|  | 15800492 |
|  | 15802498 |
|  | 15814701 |
|  | 15825075 |
|  | 15826936 |
|  | 15831701 |
|  | 15845478 |
|  | 15845623 |
|  | 15863613 |
|  | 1587308 |
|  | 15880761 |
|  | 15899942 |
|  | 15905327 |
|  | 15905495 |
|  | 15905628 |
|  | 15908405 |
|  | 15910760 |
|  | 15940362 |
|  | 15947070 |
|  | 15956054 |
|  | 15976028 |
|  | 15989949 |
|  | 1601170 |
|  | 16022585 |
|  | 16023831 |
|  | 16026997 |
|  | 16040151 |
|  | 16046706 |
|  | 16052598 |
|  | 16081806 |
|  | 16085472 |
|  | 16095565 |
|  | 1611993 |
|  | 16141072 |
|  | 16141073 |
|  | 16143138 |
|  | 16192425 |
|  | 16254126 |
|  | 16284237 |
|  | 16285960 |
|  | 1628813 |
|  | 1628826 |
|  | 16293680 |
|  | 16310049 |
|  | 16318580 |
|  | 16323703 |
|  | 16388483 |
|  | 16397521 |
|  | 16418171 |
|  | 16423921 |
|  | 16455769 |
|  | 16466693 |
|  | 16489506 |
|  | 1649220 |
|  | 16554296 |
|  | 16567518 |
|  | 16571868 |
|  | 16581004 |
|  | 16611629 |
|  | 16651528 |
|  | 16670770 |
|  | 16677721 |
|  | 16707807 |
|  | 16710471 |
|  | 16718380 |
|  | 16785562 |
|  | 16791879 |
|  | 16815711 |
|  | 16843000 |
|  | 16844679 |
|  | 16844762 |
|  | 16914507 |
|  | 16939807 |
|  | 16990136 |
|  | 17003332 |
|  | 17010015 |
|  | 17050710 |
|  | 17077645 |
|  | 17086187 |
|  | 17141199 |
|  | 17142776 |
|  | 1729694 |
|  | 1794049 |
|  | 1794058 |
|  | 1851139 |
|  | 1889804 |
|  | 1889815 |
|  | 2106569 |
|  | 2243807 |
|  | 2466396 |
|  | 2563387 |
|  | 2565278 |
|  | 2575221 |
|  | 2592764 |
|  | 2798426 |
|  | 2965020 |
|  | 3262872 |
|  | 3263439 |
|  | 7490100 |
|  | 7507352 |
|  | 7508917 |
|  | 7513199 |
|  | 7513204 |
|  | 7515712 |
|  | 7522168 |
|  | 7524893 |
|  | 7536685 |
|  | 7538528 |
|  | 7567467 |
|  | 7590746 |
|  | 7599173 |
|  | 7670492 |
|  | 7719983 |
|  | 7720571 |
|  | 7750122 |
|  | 7759110 |
|  | 7768607 |
|  | 7769130 |
|  | 7780034 |
|  | 7790819 |
|  | 7826951 |
|  | 7850809 |
|  | 7851897 |
|  | 7890367 |
|  | 7919654 |
|  | 7919658 |
|  | 7930875 |
|  | 7931061 |
|  | 7953712 |
|  | 7959747 |
|  | 7959868 |
|  | 7964164 |
|  | 7972007 |
|  | 7974717 |
|  | 8043948 |
|  | 8043949 |
|  | 8050491 |
|  | 8054487 |
|  | 8081873 |
|  | 8100556 |
|  | 8127368 |
|  | 8131749 |
|  | 8138038 |
|  | 8148154 |
|  | 8160012 |
|  | 8177505 |
|  | 8207122 |
|  | 8307620 |
|  | 8313907 |
|  | 8376770 |
|  | 8418070 |
|  | 8464030 |
|  | 8499659 |
|  | 8521822 |
|  | 8536793 |
|  | 8551238 |
|  | 8575820 |
|  | 8613694 |
|  | 8631753 |
|  | 8649829 |
|  | 8666800 |
|  | 8666893 |
|  | 8670868 |
|  | 8675203 |
|  | 8676061 |
|  | 8676083 |
|  | 8698502 |
|  | 8703029 |
|  | 8757508 |
|  | 8757858 |
|  | 8770556 |
|  | 8836549 |
|  | 8879230 |
|  | 8910279 |
|  | 8911153 |
|  | 8920166 |
|  | 8940342 |
|  | 8960823 |
|  | 9000479 |
|  | 9000540 |
|  | 9034153 |
|  | 9039941 |
|  | 9058758 |
|  | 9069127 |
|  | 9075932 |
|  | 9084435 |
|  | 9165025 |
|  | 9168824 |
|  | 9169772 |
|  | 9169855 |
|  | 9227345 |
|  | 9237710 |
|  | 9240457 |
|  | 9277378 |
|  | 9278335 |
|  | 9281358 |
|  | 9284811 |
|  | 9284817 |
|  | 9307269 |
|  | 9344502 |
|  | 9351462 |
|  | 9353074 |
|  | 9359411 |
|  | 9379832 |
|  | 9403069 |
|  | 9417815 |
|  | 9430546 |
|  | 9463396 |
|  | 9466312 |
|  | 9493504 |
|  | 9566762 |
|  | 9605877 |
|  | 9637508 |
|  | 9645618 |
|  | 9659221 |
|  | 9675420 |
|  | 9688988 |
|  | 9693973 |
|  | 9708822 |
|  | 9730900 |
|  | 9769156 |
|  | 9780277 |
|  | 9806851 |
|  | 9815267 |
|  | 9839821 |
|  | 9850165 |
|  | 9854025 |
|  | 9862349 |
|  | 9862671 |
|  | 9875345 |
|  | 9892616 |
|  | 9916891 |
|  | 9918916 |
|  | 9933112 |
|  | 9950766 |
|  | 10358063 |
|  | 10657672 |
|  | 10671302 |
|  | 10775602 |
|  | 10925276 |
|  | 10964543 |
|  | 11448119 |
|  | 11500818 |
|  | 11687509 |
|  | 11689614 |
|  | 11716039 |
|  | 11733366 |
|  | 11751408 |
|  | 11758653 |
|  | 11774563 |
|  | 11777983 |
|  | 11781191 |
|  | 11788581 |
|  | 11792588 |
|  | 11794009 |
|  | 11801594 |
|  | 11818668 |
|  | 11820460 |
|  | 11847482 |
|  | 11855786 |
|  | 11858187 |
|  | 11884401 |
|  | 11884403 |
|  | 11889177 |
|  | 11914754 |
|  | 11918227 |
|  | 11919083 |
|  | 11949822 |
|  | 11950481 |
|  | 11959895 |
|  | 11960393 |
|  | 11961304 |
|  | 11966578 |
|  | 11984595 |
|  | 11992567 |
|  | 12000745 |
|  | 12012622 |
|  | 12027404 |
|  | 12031914 |
|  | 12032172 |
|  | 12036196 |
|  | 12038454 |
|  | 12067898 |
|  | 12070032 |
|  | 12080442 |
|  | 12091348 |
|  | 12096924 |
|  | 12096927 |
|  | 12107724 |
|  | 12114287 |
|  | 12117737 |
|  | 12117921 |
|  | 12117953 |
|  | 12121679 |
|  | 12140751 |
|  | 12149213 |
|  | 12150965 |
|  | 12151314 |
|  | 12165085 |
|  | 12176919 |
|  | 12183057 |
|  | 12185451 |
|  | 12187073 |
|  | 12198224 |
|  | 12201209 |
|  | 12202945 |
|  | 12218157 |
|  | 12219016 |
|  | 12220549 |
|  | 12226829 |
|  | 12235153 |
|  | 12239630 |
|  | 12297113 |
|  | 12297725 |
|  | 12359225 |
|  | 12359431 |
|  | 12371985 |
|  | 12372336 |
|  | 12382118 |
|  | 12383455 |
|  | 12391243 |
|  | 12393446 |
|  | 12393542 |
|  | 12412787 |
|  | 12413693 |
|  | 12419823 |
|  | 12430875 |
|  | 12431817 |
|  | 12445202 |
|  | 12445803 |
|  | 12451269 |
|  | 12453469 |
|  | 12468916 |
|  | 12472176 |
|  | 12477932 |
|  | 12482836 |
|  | 12483530 |
|  | 12493411 |
|  | 12495721 |
|  | 12509497 |
|  | 12517591 |
|  | 12517814 |
|  | 12519862 |
|  | 12540635 |
|  | 12553555 |
|  | 12559950 |
|  | 12560873 |
|  | 12571160 |
|  | 12584029 |
|  | 12585340 |
|  | 12589429 |
|  | 12592380 |
|  | 12594059 |
|  | 12606524 |
|  | 12615367 |
|  | 12626585 |
|  | 12629515 |
|  | 12634650 |
|  | 12637697 |
|  | 12653788 |
|  | 12660820 |
|  | 12660878 |
|  | 12663465 |
|  | 12668157 |
|  | 12681965 |
|  | 12690205 |
|  | 12709682 |
|  | 12713584 |
|  | 12714254 |
|  | 12714267 |
|  | 12719374 |
|  | 12720537 |
|  | 12727794 |
|  | 12727841 |
|  | 12727948 |
|  | 12727959 |
|  | 12736743 |
|  | 12742994 |
|  | 12745542 |
|  | 12748876 |
|  | 12754410 |
|  | 12760309 |
|  | 12768442 |
|  | 12770686 |
|  | 12771987 |
|  | 12789531 |
|  | 12792728 |
|  | 12794182 |
|  | 12799618 |
|  | 12802423 |
|  | 12810629 |
|  | 12815949 |
|  | 12819039 |
|  | 12829659 |
|  | 12829785 |
|  | 12830005 |
|  | 12832117 |
|  | 12832326 |
|  | 12839942 |
|  | 12840146 |
|  | 12843190 |
|  | 12843357 |
|  | 12846758 |
|  | 12850808 |
|  | 12853948 |
|  | 12857677 |
|  | 12857678 |
|  | 12870175 |
|  | 12871882 |
|  | 12876073 |
|  | 12883476 |
|  | 12888915 |
|  | 12900415 |
|  | 12901869 |
|  | 1291290 |
|  | 12923493 |
|  | 12926107 |
|  | 12928051 |
|  | 12932839 |
|  | 12934185 |
|  | 12937023 |
|  | 12940515 |
|  | 12958150 |
|  | 12960348 |
|  | 12967650 |
|  | 12969979 |
|  | 13678786 |
|  | 14508181 |
|  | 14508221 |
|  | 14512079 |
|  | 14521945 |
|  | 14523169 |
|  | 14532843 |
|  | 14557255 |
|  | 14557477 |
|  | 14582815 |
|  | 14585094 |
|  | 14604894 |
|  | 14607690 |
|  | 14634582 |
|  | 14634584 |
|  | 14643032 |
|  | 14654926 |
|  | 14676217 |
|  | 14684705 |
|  | 14688371 |
|  | 14691340 |
|  | 14711458 |
|  | 14718574 |
|  | 14733913 |
|  | 14748439 |
|  | 14967376 |
|  | 14970177 |
|  | 14981536 |
|  | 15016829 |
|  | 15019283 |
|  | 15025214 |
|  | 15028733 |
|  | 15034082 |
|  | 15041717 |
|  | 15041721 |
|  | 15044662 |
|  | 15051507 |
|  | 15070705 |
|  | 15070960 |
|  | 15072962 |
|  | 15075299 |
|  | 15086488 |
|  | 15100309 |
|  | 15118287 |
|  | 15121272 |
|  | 15152368 |
|  | 15153787 |
|  | 15158360 |
|  | 15166129 |
|  | 15167972 |
|  | 15172007 |
|  | 15180970 |
|  | 15180973 |
|  | 15193488 |
|  | 15193763 |
|  | 15194822 |
|  | 15203561 |
|  | 15209388 |
|  | 15220185 |
|  | 15223612 |
|  | 15230289 |
|  | 15256272 |
|  | 15256287 |
|  | 15273081 |
|  | 15273551 |
|  | 15284113 |
|  | 15292466 |
|  | 15299003 |
|  | 15304054 |
|  | 15304377 |
|  | 15308667 |
|  | 15308875 |
|  | 15313471 |
|  | 15322060 |
|  | 15331795 |
|  | 15341921 |
|  | 15345683 |
|  | 15373762 |
|  | 15375172 |
|  | 15375575 |
|  | 15377473 |
|  | 15383370 |
|  | 15448117 |
|  | 15452323 |
|  | 15462469 |
|  | 15462470 |
|  | 15466088 |
|  | 15467541 |
|  | 15469436 |
|  | 15472205 |
|  | 15494357 |
|  | 15497456 |
|  | 15507390 |
|  | 15531184 |
|  | 15550378 |
|  | 15561952 |
|  | 15564333 |
|  | 15570310 |
|  | 15582581 |
|  | 15591791 |
|  | 15596840 |
|  | 15604420 |
|  | 15609131 |
|  | 15609321 |
|  | 15610507 |
|  | 15610530 |
|  | 15616014 |
|  | 15619425 |
|  | 15638425 |
|  | 15645209 |
|  | 15661740 |
|  | 15664789 |
|  | 15665272 |
|  | 15677734 |
|  | 15688424 |
|  | 15710474 |
|  | 15712220 |
|  | 15718495 |
|  | 15718915 |
|  | 15733644 |
|  | 15737987 |
|  | 15741245 |
|  | 15745939 |
|  | 15746456 |
|  | 15756054 |
|  | 15769552 |
|  | 15780075 |
|  | 15784966 |
|  | 15790728 |
|  | 15793269 |
|  | 15816827 |
|  | 15831279 |
|  | 15831362 |
|  | 15837947 |
|  | 15845864 |
|  | 15847080 |
|  | 15847702 |
|  | 15859365 |
|  | 15863389 |
|  | 15883744 |
|  | 15894122 |
|  | 15894558 |
|  | 15894559 |
|  | 15895091 |
|  | 15896304 |
|  | 15902127 |
|  | 15904871 |
|  | 15905620 |
|  | 15905624 |
|  | 15910501 |
|  | 15919832 |
|  | 15935883 |
|  | 15940250 |
|  | 15951664 |
|  | 15955385 |
|  | 15969253 |
|  | 15981030 |
|  | 15985687 |
|  | 15986200 |
|  | 15988318 |
|  | 16006475 |
|  | 16006970 |
|  | 16009564 |
|  | 16014026 |
|  | 16032529 |
|  | 16043358 |
|  | 16043746 |
|  | 16051899 |
|  | 16096323 |
|  | 16098388 |
|  | 16099893 |
|  | 1610348 |
|  | 16107886 |
|  | 16115611 |
|  | 16115908 |
|  | 16115919 |
|  | 16127269 |
|  | 16135414 |
|  | 16140413 |
|  | 16147857 |
|  | 16148087 |
|  | 16149059 |
|  | 16179573 |
|  | 16181755 |
|  | 16183563 |
|  | 16185356 |
|  | 16229816 |
|  | 16260731 |
|  | 16265697 |
|  | 16269662 |
|  | 16269960 |
|  | 16276011 |
|  | 16278387 |
|  | 16278864 |
|  | 16288983 |
|  | 16293576 |
|  | 16306329 |
|  | 16310043 |
|  | 16324758 |
|  | 16338121 |
|  | 16339311 |
|  | 16340466 |
|  | 16372246 |
|  | 16391840 |
|  | 16413198 |
|  | 16413199 |
|  | 16426569 |
|  | 16428383 |
|  | 16442710 |
|  | 16469407 |
|  | 16491351 |
|  | 16499908 |
|  | 16507605 |
|  | 16516857 |
|  | 16518755 |
|  | 16519772 |
|  | 16524883 |
|  | 16552338 |
|  | 16564564 |
|  | 16565838 |
|  | 16611358 |
|  | 16642549 |
|  | 16644865 |
|  | 16647242 |
|  | 16648144 |
|  | 16684268 |
|  | 16732314 |
|  | 16760905 |
|  | 16769764 |
|  | 16802343 |
|  | 16804071 |
|  | 16837641 |
|  | 16840657 |
|  | 16857895 |
|  | 16864901 |
|  | 16864905 |
|  | 16864906 |
|  | 16864908 |
|  | 16869943 |
|  | 16879054 |
|  | 16887796 |
|  | 16912152 |
|  | 16916659 |
|  | 16919469 |
|  | 16921112 |
|  | 16926159 |
|  | 16938116 |
|  | 16939660 |
|  | 16954375 |
|  | 16988089 |
|  | 16989825 |
|  | 16998242 |
|  | 17003362 |
|  | 17003778 |
|  | 17012252 |
|  | 17052299 |
|  | 17055359 |
|  | 17072955 |
|  | 17076613 |
|  | 17083041 |
|  | 17097497 |
|  | 17101288 |
|  | 17109502 |
|  | 17112887 |
|  | 17115422 |
|  | 17122961 |
|  | 17187019 |
|  | 1883960 |
|  | 2472117 |
|  | 2680567 |
|  | 2789513 |
|  | 2829354 |
|  | 2906047 |
|  | 3023045 |
|  | 3266463 |
|  | 3294161 |
|  | 3320204 |
|  | 3491322 |
|  | 3538015 |
|  | 3758081 |
|  | 7494249 |
|  | 7511596 |
|  | 7523444 |
|  | 7526541 |
|  | 7595060 |
|  | 7656270 |
|  | 7693046 |
|  | 7803268 |
|  | 7986583 |
|  | 8113688 |
|  | 8179923 |
|  | 8190832 |
|  | 8206884 |
|  | 8510564 |
|  | 8938574 |
|  | 9094636 |
|  | 9169458 |
|  | 9378998 |
|  | 9671211 |
|  | 9792375 |
|  | 9927325 |
|  | 15015143 |
|  | 15489334 |
|  | 16547804 |
|  | 16620830 |
|  | 16690518 |
|  | 16721634 |
|  | 16764962 |
|  | 16773437 |
|  | 16782174 |
|  | 16787648 |
|  | 16870193 |
|  | 16952201 |
|  | 16978071 |
|  | 16997495 |
|  | 17023074 |
|  | 17047337 |
|  | 17079331 |
|  | 17090647 |
|  | 17115186 |
|  | 17119059 |
|  | 17139467 |
|  | 17161613 |
|  | 17182550 |
|  | 17182572 |
|  | 17182950 |
|  | 17192276 |
|  | 17192581 |
|  | 17194482 |
|  | 17196171 |
|  | 17203290 |
|  | 17209781 |
|  | 17213200 |
|  | 17220301 |
|  | 17241868 |
|  | 17241887 |
|  | 17261502 |
|  | 17272771 |
|  | 17286759 |
|  | 17306245 |
|  | 17310346 |
|  | 17322283 |
|  | 17335789 |
|  | 17338374 |
|  | 17362770 |
|  | 17373716 |
|  | 17374439 |
|  | 17379849 |
|  | 17392554 |
|  | 17412556 |
|  | 17416433 |
|  | 17420140 |
|  | 17428355 |
|  | 17439892 |
|  | 17441979 |
|  | 17442294 |
|  | 17448042 |
|  | 17463301 |
|  | 17465679 |
|  | 17466952 |
|  | 17472849 |
|  | 17475670 |
|  | 17476620 |
|  | 17487763 |
|  | 17490702 |
|  | 17505301 |
|  | 17526950 |
|  | 17530721 |
|  | 17531096 |
|  | 17532054 |
|  | 17544674 |
|  | 17560462 |
|  | 17596140 |
|  | 17597660 |
|  | 17608808 |
|  | 17610900 |
|  | 17656329 |
|  | 17665434 |
|  | 17684217 |
|  | 17684929 |
|  | 17716608 |
|  | 17785926 |
|  | 2037043 |
|  | 2610854 |
|  | 3279116 |
|  | 3500852 |
|  | 7851440 |
|  | 8555185 |
|  | 9118960 |
|  | 9159484 |
|  | 10528216 |
|  | 11034416 |
|  | 11441071 |
|  | 11494166 |
|  | 11867592 |
|  | 12133972 |
|  | 12207010 |
|  | 12507769 |
|  | 12855817 |
|  | 14597766 |
|  | 14764707 |
|  | 15124018 |
|  | 16129814 |
|  | 16275384 |
|  | 16717113 |
|  | 16763563 |
|  | 16996249 |
|  | 17065508 |
|  | 17082315 |
|  | 17089190 |
|  | 17101148 |
|  | 17109471 |
|  | 17114246 |
|  | 17133451 |
|  | 17144903 |
|  | 17150369 |
|  | 17178916 |
|  | 17182564 |
|  | 17187052 |
|  | 17220181 |
|  | 17229471 |
|  | 17229673 |
|  | 17255318 |
|  | 17256754 |
|  | 17314284 |
|  | 17324158 |
|  | 17340223 |
|  | 17391979 |
|  | 17400600 |
|  | 17409491 |
|  | 17433708 |
|  | 17438336 |
|  | 17467814 |
|  | 17476359 |
|  | 17481608 |
|  | 17487773 |
|  | 17493959 |
|  | 17510220 |
|  | 17525280 |
|  | 17575466 |
|  | 17581537 |
|  | 17581588 |
|  | 17581589 |
|  | 17591976 |
|  | 17599806 |
|  | 17601518 |
|  | 17606866 |
|  | 17615358 |
|  | 17639077 |
|  | 17640882 |
|  | 17669273 |
|  | 17674319 |
|  | 2302197 |
|  | 2948184 |
|  | 3262059 |
|  | 3264160 |
|  | 7867561 |
|  | 9435302 |
|  | 9603480 |
|  | 9653168 |
|  | 16988192 |
|  | 17133350 |
|  | 17170234 |
|  | 17204394 |
|  | 17303338 |
|  | 17314490 |
|  | 17322172 |
|  | 17327367 |
|  | 17328769 |
|  | 17341596 |
|  | 17385713 |
|  | 17395896 |
|  | 17433709 |
|  | 17582939 |
|  | 17665433 |
|  | 1446077 |

---

|  |  |
| --- | --- |
| Organism | Homo sapiens {Organism urn:agi-taxid:9606} |
|  | Mus musculus {Organism urn:agi-taxid:10090} |
|  | Rattus norvegicus {Organism urn:agi-taxid:10116} |
|  | Homo sapiens |
|  | Mus musculus |
|  | Rattus norvegicus |
|  | Bos taurus |
|  | Human |
|  | mouse |
|  | murine |

---

|  |  |
| --- | --- |
| Mouse chromosome position | 5 15.7 cM |
|  | 5 17.0 cM |

---

|  |  |
| --- | --- |
| OMIM ID | 147620 |
|  | 108010 |
|  | 222100 |
|  | 266600 |
|  | 148000 |
|  | 604302 |
|  | 125853 |
|  | 166710 |
|  | OMIM:147620 |
|  | OMIM:148000 |
|  | OMIM:166710 |

---

|  |  |
| --- | --- |
| Rat chromosome position | 4q11 |

---

|  |  |
| --- | --- |
| Hugo ID | 6018 |
|  | HGNC:6018 |

---

|  |  |
| --- | --- |
| Human chromosome position | 7p15.3 |
|  | 7p21 |

---

|  |  |
| --- | --- |
| Swiss-Prot Accession | B4DVM1 |
|  | P05231 |
|  | Q75MH2 |
|  | B5MC21 |
|  | B4DNQ5 |
|  | P05231.1 |
|  | A0A0G2JGF4 |
|  | P08505 |
|  | A2RTD1 |
|  | P08505.1 |
|  | P20607 |
|  | P20607.1 |
|  | Q9UCU2 |
|  | Q9UCU3 |
|  | Q9UCU4 |
|  | Q3UCQ0 |
|  | Q8BN26 |
|  | Q8N6X1 |
|  | Q0PMN1 |
|  | Q3U8J8 |
|  | IL6\_HUMAN |
|  | IL6\_MOUSE |
|  | IL6\_RAT |
|  | Q75MH2\_HUMAN |
|  | A2RTD1\_MOUSE |

---

|  |  |
| --- | --- |
| PIR ID | A32648 |
|  | A30531 |
|  | A34247 |

---

|  |  |
| --- | --- |
| GenBank ID | NC\_000007 |
|  | XM\_011515390 |
|  | XP\_011513692 |
|  | XM\_005249745 |
|  | XP\_005249802 |
|  | NM\_000600 |
|  | NP\_000591 |
|  | NM\_001318095 |
|  | NP\_001305024 |
|  | NG\_011640 |
|  | AC073072 |
|  | AAS07539 |
|  | AF005485 |
|  | AF039224 |
|  | AF039225 |
|  | AF039226 |
|  | AF039227 |
|  | AF039228 |
|  | AF048692 |
|  | AAC05673 |
|  | AF362378 |
|  | AF372214 |
|  | AAK48987 |
|  | AY170325 |
|  | AAN75009 |
|  | CH236948 |
|  | EAL24265 |
|  | CH471073 |
|  | EAW93756 |
|  | EAW93757 |
|  | EAW93758 |
|  | CS352987 |
|  | CAL23031 |
|  | JA104583 |
|  | CCA63961 |
|  | JQ250825 |
|  | AFF18412 |
|  | KX430120 |
|  | AOP17752 |
|  | KX430126 |
|  | AOP17758 |
|  | KX430127 |
|  | AOP17759 |
|  | KX430128 |
|  | AOP17760 |
|  | KX430129 |
|  | AOP17761 |
|  | KX430130 |
|  | AOP17762 |
|  | KX430131 |
|  | AOP17763 |
|  | X04402 |
|  | CAA27990 |
|  | Y00081 |
|  | CAA68278 |
|  | A09363 |
|  | CAA00839 |
|  | AA721478 |
|  | AK298013 |
|  | BAG60317 |
|  | AK298077 |
|  | BAG60365 |
|  | AK301141 |
|  | BAG62733 |
|  | BC015511 |
|  | AAH15511 |
|  | BT019748 |
|  | AAV38553 |
|  | BT019749 |
|  | AAV38554 |
|  | CD013918 |
|  | CR450296 |
|  | CAG29292 |
|  | HY076725 |
|  | M14584 |
|  | AAA52728 |
|  | M18403 |
|  | AAA52729 |
|  | M29150 |
|  | AAA59154 |
|  | M54894 |
|  | AAC41704 |
|  | S56892 |
|  | AAD13886 |
|  | X04403 |
|  | CAA27991 |
|  | X04430 |
|  | CAA28026 |
|  | X04602 |
|  | CAA28268 |
|  | P05231 |
|  | NC\_000071 |
|  | NM\_031168 |
|  | NP\_112445 |
|  | NM\_001314054 |
|  | NP\_001300983 |
|  | AC112933 |
|  | CH466524 |
|  | EDL37257 |
|  | EDL37258 |
|  | M20572 |
|  | AAA39302 |
|  | M24221 |
|  | AAA68814 |
|  | M36996 |
|  | X51457 |
|  | CAA35824 |
|  | AK089780 |
|  | AK150440 |
|  | BAE29562 |
|  | AK152189 |
|  | BAE31020 |
|  | BC132458 |
|  | AAI32459 |
|  | BC138766 |
|  | AAI38767 |
|  | BC145409 |
|  | DQ788722 |
|  | ABG81953 |
|  | J03783 |
|  | AAA39301 |
|  | X06203 |
|  | CAA29560 |
|  | X54542 |
|  | CAA38411 |
|  | P08505 |
|  | NC\_005103 |
|  | NM\_012589 |
|  | NP\_036721 |
|  | AC\_000072 |
|  | AABR07059069 |
|  | AAHX01027501 |
|  | CH474057 |
|  | EDL86435 |
|  | M26745 |
|  | AAA41430 |
|  | M26744 |
|  | AAA77659 |
|  | P20607 |
|  | XM\_011515391 |
|  | XP\_011513693 |
|  | NC\_018918 |
|  | AMYH02015349 |
|  | DQ891463 |
|  | ABM82389 |
|  | DQ894639 |
|  | ABM85565 |
|  | AC\_000027 |
|  | AAHY01043243 |
|  | AC\_000139 |
|  | ABBA01056696 |
|  | XM\_005249746 |
|  | XP\_005249803 |
|  | AC\_000068 |
|  | AACC02000007 |
|  | AABR06028560 |
|  | NT\_007819 |
|  | NT\_079592 |
|  | NW\_001839003 |
|  | NW\_923240 |
|  | AC\_000050 |
|  | CR590965 |
|  | CR626263 |
|  | Q75MH2 |
|  | Q8N6X1 |
|  | NT\_162294 |
|  | NW\_001030787 |
|  | Q0PMN1 |
|  | Q3U8J8 |
|  | NW\_047683 |
|  | NW\_001084822 |
|  | NW\_000226 |
|  | NT\_086703 |
|  | 124350 |
|  | 204916 |
|  | 204920 |
|  | 110671381 |
|  | 123784328 |
|  | 123792630 |
|  | 124348 |
|  | 124376266 |
|  | 309410 |
|  | 387386 |
|  | 52702 |
|  | 52728 |
|  | 581860 |
|  | 74212560 |
|  | 74219584 |
|  | 870699 |
|  | 119614162 |
|  | 119614164 |
|  | 123995927 |
|  | 124347 |
|  | 13899131 |
|  | 15930149 |
|  | 186352 |
|  | 23833 |
|  | 23835 |
|  | 29495 |
|  | 306910 |
|  | 306911 |
|  | 307063 |
|  | 32674 |
|  | 33850 |
|  | 41474122 |
|  | 4261586 |
|  | 47496539 |
|  | 490070 |
|  | 51095021 |
|  | 54696362 |
|  | 54696364 |
|  | 74729191 |
|  | 74739144 |
|  | 119614163 |
|  | 123981120 |
|  | 148705310 |
|  | 148705311 |
|  | 149031445 |
|  | 115304889 |
|  | 124346 |
|  | 2194 |
|  | AAI23578 |
|  | BC123577 |
|  | CAA40572 |
|  | NC\_007302 |
|  | NM\_173923 |
|  | NP\_776348 |
|  | NW\_001494879 |
|  | P26892 |
|  | X57317 |
|  | NT\_039300 |
|  | BAC40960 |
|  | BC027978 |
|  | AAH27978 |
|  | NW\_043536 |
|  | NT\_007819.11 |

---

|  |  |
| --- | --- |
| Swiss-Prot ID | IL6\_HUMAN |
|  | IL6\_MOUSE |
|  | IL6\_RAT |
|  | B4DVM1\_HUMAN |
|  | A2RTD1\_MOUSE |
|  | Q75MH2\_HUMAN |

---

|  |  |
| --- | --- |
| Cell Localization | Secreted |
|  | Extracellular |

---

|  |  |
| --- | --- |
| Ensembl ID | ENSG00000136244 |
|  | ENSP00000258743.5 |
|  | ENST00000258743.10 |
|  | ENSP00000385043.1 |
|  | ENST00000407492.5 |
|  | ENSP00000385675.1 |
|  | ENST00000404625.5 |
|  | ENSMUSG00000025746 |
|  | ENSMUSP00000143544.1 |
|  | ENSMUST00000195978.4 |
|  | ENSMUSP00000026845.7 |
|  | ENSMUST00000026845.11 |
|  | ENSRNOG00000010278 |
|  | ENSRNOP00000013732.6 |
|  | ENSRNOT00000013732.6 |
|  | ENST00000258743.9 |
|  | ENSP00000258743 |
|  | ENST00000258743 |
|  | ENSP00000385675 |
|  | ENST00000404625 |
|  | ENSP00000385043 |
|  | ENST00000407492 |
|  | ENSMUSP00000026845 |
|  | ENSMUST00000026845 |
|  | ENSRNOP00000013732 |
|  | ENSRNOT00000013732 |
|  | ENSP00000405994 |
|  | ENST00000420258 |

---

|  |  |
| --- | --- |
| MGI ID | MGI:96559 |
|  | 96559 |

---

|  |  |
| --- | --- |
| RGD ID | 2901 |

---

|  |  |
| --- | --- |
| Unigene ID | Mm.1019 |
|  | Rn.9873 |
|  | Hs.654458 |
|  | Hs.512234 |
|  | Bt.3686 |
|  | Hs.93913 |

---

|  |  |
| --- | --- |
| Homologene ID | 502 |

---

|  |  |
| --- | --- |
| Shape | Rhomb |

---

|  |  |
| --- | --- |
| IPI ID | IPI00133702 |
|  | IPI00007793 |
|  | IPI00201260 |
|  | IPI00878107 |
|  | IPI00167115 |
|  | IPI00878334 |
|  | IPI00878660 |
|  | IPI00877815 |
|  | IPI00877634 |

---

|  |  |
| --- | --- |
| Microarray ID | 102218\_at |
|  | A\_32\_P189757 |
|  | I\_1960905 |
|  | A\_32\_P67060 |
|  | I\_1960906 |
|  | 1369191\_at |
|  | M26744\_at |
|  | M26745cds\_s\_at |
|  | A\_43\_P11475 |
|  | NM\_012589 |
|  | 1450297\_at |
|  | x54542\_s\_at |
|  | Msa.849.0\_at |
|  | A\_51\_P217218 |
|  | NM\_031168 |
|  | M14584 >2 |
|  | M14584 >1 |
|  | 38299\_at |
|  | 205207\_at |
|  | X04602\_s\_at |
|  | Y00081\_s\_at |
|  | A\_23\_P71037 |
|  | I\_929482 |
|  | A\_14\_P133151 |
|  | A\_23\_P305818 |

---

|  |  |
| --- | --- |
| Homo sapiens Chromosome position | 7p21 |

---

|  |  |
| --- | --- |
| Ensembl Transcript ID | ENST00000258743 |
|  | ENST00000404625 |

---

|  |  |
| --- | --- |
| FunctionalClass | cytokine |
|  | interleukin-6 receptor binding |
|  | protein binding |
|  | growth factor |

---

|  |  |
| --- | --- |
| Bos taurus Chromosome position | 4 |

---

|  |  |
| --- | --- |
| Mus musculus Chromosome position | 5 17.0 cM |

---

|  |  |
| --- | --- |
| Proteome Subcellular Localization | Extracellular (excluding cell wall) |
|  | Cytoplasmic |

---

|  |  |
| --- | --- |
| Compartment | RNA |

---

|  |  |
| --- | --- |
| KEGG pathway | Hematopoietic cell lineage |
|  | Cytokine-cytokine receptor interaction |
|  | Jak-STAT signaling pathway |
|  | Toll-like receptor signaling pathway |

---

|  |  |
| --- | --- |
| RefSeq ACCNUM | NP\_000591 |

---

|  |  |
| --- | --- |
| Rattus norvegicus Chromosome position | 4q11 |

---

|  |  |
| --- | --- |
| Ensembl Gene ID | ENSG00000136244 |

---

|  |  |
| --- | --- |
| Keyword | desmoplakin |
|  | map kinase |
|  | hnRNA |
|  | growth factor |
|  | cytokine receptor |
|  | exotoxin |
|  | interleukin-1 receptor ligand |
|  | acetyltransferase |
|  | desmoplakins |
|  | map kinases |
|  | binding activity |
|  | tumor necrosis factor |
|  | RNA |
|  | cytokine activity |

---

|  |  |
| --- | --- |
| LocatorString | pmquery:curcumin+prostate+cancer |
|  | pmquery:curcumin prostate cancer |
|  | digtext:C:\Documents and Settings\yuryev\My Documents\Customers\Kristina Hettne\CPRS input entities.txt |

---

|  |  |
| --- | --- |
| Proteome Organismal Role | Cell fate specification |
|  | Bone Development and Maintenance |
|  | Anti-pathogen response |
|  | Cell-to-cell signalling |
|  | Control of Cell Proliferation |

---
